# NADPH Oxidase 5 (NOX5) Overexpression Promotes Endothelial Dysfunction via Cell Apoptosis, Migration, and Metabolic Alterations in Human Brain Microvascular Endothelial Cells (hCMEC/D3)

**DOI:** 10.3390/antiox11112147

**Published:** 2022-10-29

**Authors:** Javier Marqués, Joaquín Fernández-Irigoyen, Elena Ainzúa, María Martínez-Azcona, Adriana Cortés, Carmen Roncal, Josune Orbe, Enrique Santamaría, Guillermo Zalba

**Affiliations:** 1 Navarra Institute for Health Research (IdiSNA), 31008 Pamplona, Spain; 2 Department of Biochemistry and Genetics, University of Navarra, 31008 Pamplona, Spain; 3 Clinical Neuroproteomics Unit, Navarrabiomed, Hospital Universitario de Navarra (HUN), 31008 Pamplona, Spain; 4 Laboratory of Atherothrombosis, Program of Cardiovascular Diseases, Cima Universidad de Navarra, 31008 Pamplona, Spain; 5 Centro de Investigación Biomédica en Red Cardiovascular—CIBERCV, 28029 Madrid, Spain; 6 RICORS-Cerebrovascular Diseases, Instituto de Salud Carlos III, 28029 Madrid, Spain

**Keywords:** NADPH oxidase 5, endothelial cells, endothelial dysfunction, cell proliferation, cell migration, mitochondrial dysfunction, cardiovascular diseases

## Abstract

NADPH oxidases (NOX) constitute the main reactive oxygen species (ROS) source in blood vessels. An oxidative stress situation due to ROS overproduction can lead into endothelial dysfunction, a molecular mechanism that precedes cardiovascular diseases (CVDs) such as atherosclerosis, myocardial infarction, and stroke. NOX5 is the last discovered member of the NOX family, studied in a lesser extent due to its absence in the rodent genome. Our objective was to describe the phenotypic alterations produced by an oxidative stress situation derived from NOX5 overexpression in an endothelial in vitro model. The in vitro model consists of the hCMEC/D3 cell line, derived from brain microvascular endothelium, infected with a recombinant NOX5-β adenovirus. After an initial proteomic analysis, three phenotypic alterations detected in silico were studied: cell proliferation and apoptosis, general and mitochondrial metabolism, and migration capacity. NOX5 infection of hCMEC/D3 generates a functional protein and an increase in ROS production. This model produced changes in the whole cell proteome. The in silico analysis together with in vitro validations demonstrated that NOX5 overexpression inhibits proliferation and promotes apoptosis, metabolic alterations and cell migration in hCMEC/D3 cells. NOX5 overexpression in endothelial cells leads to phenotypic changes that can lead to endothelial dysfunction, the onset of atherosclerosis, myocardial infarction, and stroke.

## 1. Introduction

The endothelium is composed by a cell monolayer involved in vascular homeostasis as it regulates vascular tone, leukocyte extravasation, and vessel permeability among other key functions [1]. These functions and endothelial homeostasis are strictly regulated by paracrine molecules, as nitric oxide (NO) [1,2] and interleukins [3,4]; or endocrine physiological stimuli, as Angiotensin II [3]. The dysregulation of endothelial cells underlies the molecular mechanisms of several cardiovascular diseases (CVDs) such as atherosclerosis. In fact, endothelial dysfunction has been proposed as an early predictor of cardiovascular risk, as it correlates with the main risk factors of these diseases: hypertension, hypercholesterolemia, diabetes mellitus, and chronic smoking [5].

One of the main molecular mechanisms involved in endothelial dysfunction is oxidative stress [1,3], which consists of a disbalance of redox homeostasis due to ROS overproduction or antioxidant systems depletion. Increased ROS production may be due to several pro-oxidant enzymes including uncoupled endothelial nitric oxide synthase, xanthine oxidase, mitochondrial oxidases, and NADPH oxidases (NOXs), these later being the only professional ROS producer enzymes [6]. NOX expression in the vascular wall has been extensively linked to CVDs and it has been proposed as a therapeutic target for atherosclerosis [7,8] and stroke [9,10]. Within this group of enzymes, NADPH oxidase 5 is directly regulated by intracellular calcium levels ([Ca^++^]) due to its EF-hand domains, but also by CVD-related stimuli such as glucose, angiotensin II, or endothelin 1. So far, NOX5 is the least studied NOX due to its absence in the rodent genome, despite its potential role in CVDs [11].

NOX5 expression in the vessel wall is involved in several inflammation processes that may promote atherothrombosis. Thus, lysophosphatidylcholine, a component of oxidized low-density lipoproteins promotes NOX5 activation via [Ca^++^] increase in human aortic endothelial cells (HAEC) [12]. In addition, in immortalized HAEC, NOX5 overexpression favors cyclo-oxygenase-2 (COX-2) expression and activity, and Prostaglandin E_2_ (PGE_2_) production, a prostaglandin involved in the atheroma plaque development [13]. Nevertheless, the exact role of NOX5 in atherothrombosis remains unclear. Human studies revealed that endothelial NOX5 expression at mRNA and protein levels is directly related with coronary early lesions [14], while a NOX5 knock-out rabbit model showed increased aortic plaque formation [15], suggesting a protective role.

Interestingly, NOX5 has been recently related with aging and stroke. Endothelial NOX5 expression in a conditional in vivo model led to memory loss in elderly mice caused by an increased blood-brain-barrier (BBB) permeability [16]. In another knock in model, NOX5 expression in endothelial and circulating cells produced higher infarct size and worsened physical stroke outcomes. This pathophysiological role was attributed to the post-reoxygenation phase, as brain recanalization leads to NOX5 activation and enhanced ROS production, causing endothelial damage [17]. This NOX5-derived endothelial disfunction was also observed in a diabetic retinopathy model, increasing vascular permeability and angiogenic and inflammatory factors overexpression [18]. 

These data suggest that NOX5 plays a key role in the endothelial dysfunction that precedes CVD, and more specifically the atherothrombotic process. Although it has been described as a proinflammatory, our hypothesis is that NOX5 may favor other molecular mechanisms of endothelial dysfunction. In the present project we demonstrated that NOX5 overexpression in a brain microvascular endothelial cell line (hCMEC/D3) promotes phenotypic alterations including (i) apoptosis activation and cell proliferation inhibition; (ii) increased cellular migration; and (iii) cell energy deregulation. These alterations may favor at this point the atherothrombotic process, thus worsening cardiovascular outcomes, including stroke.

## 2. Materials and Methods

### 2.1. Cell Culture

Immortalized human brain microvascular endothelial cells (hCMEC/D3) were purchased from Merck-Millipore (SCC066, Merck KGaA^®^, Darmstadt, Germany). Cells were maintained with EndoGRO-MV Complete Culture Media Kit^®^ (SCME004, Merck KGaA^®^) at 37 °C and 5% CO_2_. Culture media kit contained 0.2% EndoGRO-LS Supplement, 5 ng/mL recombinant human endothelial growth factor, 10 mM L-Glutamine, 1 μg/mL Hydrocortisone Hemisuccinate, 0.75 U/mL Heparin Sulfate, 50 μg/mL ascorbic acid and 5% fetal bovine serum. This medium was supplemented with 0.5% streptomycin-penicillin (15140148 Gibco^TM^, Thermo Fisher Scientific Inc^®^, Waltham, MA, USA) and 0.5% gentamycin (15750060, Gibco^TM^). The medium was refreshed every 48–72 h and cells were trypsinized weekly with Trypsin-EDTA^®^ 0.05% (25300054, Gibco^TM^). Culture flasks and plates were previously coated adding Rat tail collagen type I (08-115, Merck KGaA^®^) diluted 1:6 in PBS (10010023, Gibco^TM^) for at least 1 h at room temperature. After that time the solution was removed, and the flasks and plates were air-dried for at least another hour. All the assays with the hCMEC/D3 cell line were performed with cells reaching a 95–100% confluence, a key feature for endothelial cells homeostasis and function.

A recombinant adenovirus was used to generate a NOX5-β overexpression in vitro model. This adenovirus codifies for human NOX5-β cDNA, and after preliminary studies, a multiplicity of infection (MOI) 100 was established as ideal for this in vitro system. A GFP-encoding adenovirus was used as a control condition. A solution containing the adequate adenovirus quantity was prepared in Vascular Cell Basal Medium (PCS-100-030, ATCC^®^, Manassas, VA, USA) supplemented with 2% fetal bovine serum (26140079, Gibco^TM^), 0.5% Streptomycin-penicillin (15140148, Gibco^TM^), and 0.5% gentamycin (15750060, Gibco^TM^). The cell medium was removed and this solution was added for 3 h until it was replaced with fresh medium for 9 h (12 h of infection), 21 h (24 h of infection), or 45 h (48 h of infection) as previously described by our group in transcriptomic array analysis of Human Aortic Endothelial Cells (PCS-100-011, ATCC^®^) [19].

### 2.2. Proteomic Analysis

For protein samples extraction 500,000 hCMEC/D3 cells per well were seeded in 6-well format collagen precoated plates. Cells were infected for 24 h, detached and centrifuged. The cell pellet was homogenized in a lysis buffer composed by 7 M urea, 2 M thiourea, and 50 mM DTT, containing protease and phosphatase inhibitors. Samples were then centrifuged at 100,000× *g* for 1 h at 15 °C, and protein concentration was quantified using Bradford Assay (5000001, Bio-Rad^®^, Hercules, CA, USA). Extracts were diluted in Laemmli sample buffer (1610747, BioRad^®^) and loaded into a 0.75 mm thick polyacrylamide gel with a 4% stacking gel casted over a 12.5% resolving gel. The run was stopped as soon as the front entered 3 mm into the resolving gel so that the whole proteome became concentrated in the stacking/resolving gel interface. Bands were stained with Coomassie Brilliant Blue, excised from the gel, and protein enzymatic cleavage was carried out with trypsin (Promega^®^ V5071, Madison, WI, USA; 1:20, *w*/*w*) at 37 °C for 16 h as previously described [20]. Purification and concentration of peptides were measured using C18 Zip Tip Solid Phase Extraction (C5737, Merck KGaA^®^). Peptide mixtures were separated by reverse phase chromatography using an UltiMate 3000 UHLPC System (Thermo Scientific, Rockford, IL, USA) fitted with an Aurora packed emitter column (25 cm x 75 µm ID, 1.6 µm C18, Ionopticks^®^, Australia). Samples were first loaded for desalting and concentration into an Acclaim PepMap column (0.5 cm × 300 µm ID, 5 µm C18, ThermoFisher^®^) packed with the same chemistry as the separating column. Mobile phases were 100% water 0.1% formic acid (buffer A) and 100% Acetonitrile 0.1% formic acid (buffer B). A column gradient was developed in a 120 min two step gradient from 5% B to 20% B in 90 min and 20% B to 32% B in 30 min. The column was equilibrated in 95% B for 10 min and 5% B for 20 min. During all processes, precolumn was in line with the column and flow maintained all along the gradient at 300 nl/min. The column temperature was maintained at 40 °C using an integrated column oven (PRSO-V2, Sonation^®^, Biberach, Germany) and interfaced online with the Orbitrap Exploris 480 MS. Spray voltage were set to 2 kV, funnel RF level at 40, and heated capillary temperature at 300 °C. For DDA experiments full mass spectrometry (MS) resolutions were set to 1,200,000 at m/z 200 and full MS AGC target was set to Standard with an IT mode Auto. Mass range was set to 375–1500. AGC target value for fragment spectra was set to standard with a resolution of 15,000 and 3 s for cycle time. Intensity threshold was kept at 8E3. Isolation width was set at 1.4 m/z. Normalized collision energy was set at 30%. All data were acquired in centroid mode using positive polarity and peptide match was set to off, and isotope exclusion was on. 

Raw files were processed with MaxQuant [21] v1.6.17.0 using the integrated Andromeda Search engine [22]. All data were searched against a target/decoy version of the mouse Uniprot Reference Proteome without isoforms (55,366 entries) with March 2021 release. First, search peptide tolerance was set to 20 ppm, and main search peptide tolerance was set to 4.5 ppm. Fragment mass tolerance was set to 20 ppm. Trypsin was specified as an enzyme, cleaving after all lysine and arginine residues and allowing up to two missed cleavages. Carbamidomethylation of cysteine was specified as fixed modification and peptide N-terminal acetylation, oxidation of methionine, deamidation of asparagine, and glutamine and pyro-glutamate formation from glutamine and glutamate were considered variable modifications with a total of 2 variable modifications per peptide. “Maximum peptide mass” were set to 7500 Da, the “modified peptide minimum score” and “unmodified peptide minimum score” were set to 25 and everything else was set to the default values, including the false discovery rate limit of 1% on both the peptide and protein levels. The Perseus software (version 1.6.14.0) [23,24] was used for statistical analysis and data visualization.

### 2.3. Quantitative PCR (qPCR)

For RNA samples generation, 300,000 cells were seeded in 6-well collagen-precoated plates overnight. The day after, cells were infected for 12, 24, or 48 h, the cell medium was removed, and the wells were washed once with ice-cold PBS (10010023, Gibco^TM^). After that, 1 mL of TRIzol (15595026, Thermo Scientific^®^) was added per well, and RNA extraction was performed using organic compounds. Each RNA sample was obtained in 20 μL of DEPC-treated water (AM9915G, Thermo Scientific^®^), and quantified using a Nanodrop ND-1000 spectrophotometer (Thermo Scientific^®^). One μg of RNA underwent retro transcription using SuperScript III VILO cDNA Synthesis Kit (18080085, Thermo Scientific^®^). PCR reactions with these cDNA samples were performed with iQ SYBR Green (1708880, Bio-Rad^®^) in an iQ5 Multicolor real-time PCR Detection System (Bio-Rad^®^). PCR results were normalized using Glyceraldehyde 3-phosphate dehydrogenase (GADH) as a housekeeping gene, performing each reaction by triplicate. Specific primers for each gene amplification (showed in Table 1) were designed and tuned by the group, ensuring specific amplification with melting curve. The PCR protocol followed was: 15 min incubation at 95 °C and 40 amplification cycles of 25 s at 95 °C, 15 s at the appropriate annealing temperature and 10 s at 72 °C. For qPCR analysis ΔΔCT was calculated, and samples were normalized with the control condition (results are shown as a fold increase relative to control).

### 2.4. Western Blotting

For intracellular protein extraction 500,000 cells/well were seeded in 6-well collagen-precoated plates. After the pertinent infection time, the medium was discarded, and cells were washed once with ice-cold PBS (10010023, Gibco^TM^). Cells were lysed in 100 μL of RIPA Buffer (1% NP-40, 150 mM NaCl, 50 mM Tris pH = 8, 0.1% SDS and 0.5% sodic deoxycholate) with protease inhibitors (11697498001, Merck KGaA^®^). Samples were sonicated and protein concentration was quantified using PierceTM BCA Protein Assay Kit (23225, ThermoFisher^®^). Firstly, 30 μg of protein was diluted in distilled water and 4× Laemmli Buffer (1610747, BioRad^®^) with 10% β-mercaptoethanol. Secondly, these samples were loaded in 10% acrylamide denaturalizing gels, and electrophoresis was performed for 1 h and 30 min at 120 V and room temperature. Thirdly, proteins were transferred to 0.45 μM pore nitrocellulose membranes (GE10600003, Merck KGaA^®^) for 1 h and 10 min at 350 mA and 4 °C. Finally, membranes were blocked in the pertinent solution, 5% non-fated milk in PBS or 1–5% BSA (A9418, Merck KGaA^®^) in 0.05% Tween-PBS, for at least 1 h at room temperature. For protein immunoblot detection overnight incubation at 4 °C was performed with the following antibodies: 1:5000 β-actin (A5441, Sigma Aldrich^®^) 5% milk in PBS, 1:500 NOX5 (191010, Abcam^®^, Waltham, MA, USA) 5% milk in 0.05% Tween-PBS, 1:500 ERM (3142S, Cell Signaling^TM^, Danvers, MA, USA) 1% BSA in 0.05% Tween-PBS and 1:500 P-ERM (Phospho-Ezrin(Thr567)/Radixin(Thr564)/Moesin(Thr558) (3141S, Cell Signaling^TM^) 1% BSA in 0.05% Tween-PBS. The day after that, membranes were washed three times with 0.05% Tween-PBS solution and incubated for 1 h at room temperature with 1:2000 secondary antibody linked to horseradish peroxidase against IgG constant fraction from mouse (NA931V, GE Healthcare, Merck KGaA^®^) or rabbit (NA934V, GE Healthcare, Merck KGaA^®^). Membranes were again washed three times and ECL Prime Western Blotting Detection Reagent^TM^ was used for visualization (GE28980926, Merck KGaA^®^). The protein expression level was normalized, dividing the signal of each specific protein by the β-actin signal of the same sample from the same initial gel. Membrane images were cropped in order to make the figures easier to understand and avoid the part of empty membrane in the images.

### 2.5. Extracellular H_2_O_2_ Production Measurement

Extracellular H_2_O_2_ production was measured in intact cells using AmpliFlu Red^TM^ (90101, Sigma Aldrich^®^) fluorescent kit. The day before the infection, 30,000 cells/well were seeded in 96-well collagen-precoated plates. NOX5 activators: 100 nM Phorbol 12-myristate 13-acetate (PMA) (P1585, Sigma Aldrich^®^), 25–100 nM Ionomycin (I0634, Sigma Aldrich^®^) and 100 nM Ang II (A9525, Sigma Aldrich^®^) were added 24 h after the infection for 15 min in Krebs–Ringer Bicarbonate Buffer (K4002, Merck KGaA^®^). After that incubation time, AmplifluRed kit reagents were added in an equal volume of Krebs Buffer. In the case of NOX inhibition, cells were incubated with 10 nM ML-090 (15172, Cayman Chemical^®^) since the 3rd hour of infection and 10 nM ML-090 was refreshed in Krebs–Ringer Bicarbonate Buffer and cells were incubated for 15 min. Fluorescence intensity was measured using a microplate fluorescence reader (PolarStar^®^, BMG Labtech, Ortenberg, Germany) at 544 nm for excitation and at 590 nm for emission measurement.

### 2.6. Intracellular Superoxide (O_2_^●−^ ) Production Detection

Dihydroethidium (DHE) (D11347, ThermoFisher^®^) was employed for intracellular O_2_^●−^ production semiquantitative detection. A total of 30,000 cells/well were seeded in 96-well collagen-precoated plates the day before infection. The day after the infection, cell medium was removed and cells were incubated with 50 μL of prewarmed Krebs buffer for 15 min, then 50 μL of a 10 μM DHE solution in Krebs buffer were added. Ten minutes after that, redfield images were obtained in a ZOE Fluorescent Cell Imager (Bio-Rad).

### 2.7. Apoptotic Levels Measurement

For apoptotic levels quantification a Caspase-Glo^®^ 3/7 assay kit (G8090, Promega) was used in intact cells. A total of 30,000 cells/well were seeded in 96-well collagen-precoated plates the day before infection. The day after, the cells were infected for 24 h, the caspase kit was used following manufacturer indications. Final luminescence was measured in a Luminoskan Ascent (ThermoFisher^®^) microplate luminometer.

### 2.8. Telomere Length Measurement by qPCR

Genomic DNA was extracted from hCMEC/D3 cells using KAPPA^®^ Express Extract kit (KK7103, Kapa Biosystems, Merck KGaA^®^). A total of 500,000 hCMEC/D3 cells were seeded in 6-well format precoated plates, and 24 h after infection with the pertinent adenovirus, cells were detached and centrifuged. Cell pellet was resuspended in 1X KAPPA Extraction Buffer, and the procedure was continued according to manufacturer indications. DNA quality was confirmed and its concentration was measured using Nanodrop ND-1000 spectrophotometer (Thermo Scientific^®^). 

Telomere length was measured as previously described [25], using specific primers for telomere and albumin amplification in a monochrome multiplex RT-qPCR reaction. For each sample measurement, 100 ng of gDNA per PCR reaction were added. Reactions were performed by triplicate. Telomere length is expressed as the ratio between Telomere copy number and Albumin copy number (single copy gene in human gDNA).

### 2.9. Real Time Proliferation Measurement

hCMEC/D3 proliferation was measured using the Real-Time Cell Analyzer xCELLigence (Agilent Technologies, Santa Clara, CA, USA). Firstly, 50,000 hCMEC/D3 cells were seeded in 24-well collagen-precoated plates. Two days after, one well was detached, and cells were counted to calculate the quantity of adenovirus necessary for well infection. Cells were infected in basal medium for 3 h and detached. The blank was made in a 16-well E-plate (05232368001, Agilent) precoated with collagen and with 50 μL of cell medium, then 10,000 hCMEC/D3-infected cells were seeded in additional 50 μL of cell medium per well. The Real-Time Cell Analyzer was placed in a cell incubator at 37 °C and 5% CO_2_. The automatic monitoring was performed every 15 min for a total time of 48 h. 

### 2.10. XFp Cell Mito Stress Assay

Several mitochondrial functional parameters were measured using Seahorse XST Analyzer^®^ (Agilent^TM^, Santa Clara, CA, USA). Briefly, 50,000 hCMEC/D3 cells were seeded in 24-well collagen-precoated plates. Two days after that, one well was detached, and cells were counted to calculate the quantity of adenovirus necessary for well infection. After 3 h of infection in basal medium, cells were detached and 12,000 cells/well were seeded in XST-specific plates precoated with collagen in culture medium. Just prior to the assay, the culture media was replaced as according to the manufacturer. Oxygen consumption rate (OCR) and extracellular consumption rate (ECAR) were measured under basal conditions or after the addition of 0.1 μM Oligomycin (mitochondrial complex V inhibitor), 1 μM Rotenone + Antimycin (complex I and III inhibitors), 1.5 μM 2-[2-[4-(trifluoromethoxy)phenyl]hydrazinylidene]-propanedinitrile (FCPP, a mitochondrial uncoupler) and 50 mM 2-DG (D-glucose analogs). Individual well values were normalized by total protein concentration.

### 2.11. Wound-Healing Assay

For cell migration capacity study, 50,000 cells /well were seeded in 24-well collagen-precoated format plates. The day after, cells were infected for 24 h. At that moment, the middle of the well was scratched, and pictures taken at 0, 24, and 32 h after creating the wound. Images were taken using Nikon SMZ18 light microscope (Nikon, Tokyo, Japan). Migration was measured as the % of wounds covered by the cells after the pertinent times, dividing the final scratch cell-free area by the initial scratch area using Image J^®^ (v 1.53) software (NIH, New York, NY, USA).

### 2.12. Statistical Analysis

Graph results are shown as violin plots or boxplots using standard error of the mean (SEM) and as mean ± SEM at tables. Firstly, data normality was studied for each group using Shapiro–Wilk or Kolmogorov–Smirnov test. When two independent groups were compared a *t*-test analysis was performed (considering data distribution), and when more than two groups were analyzed, a two-way ANOVA was performed. In the specific case of Real Time proliferation assay the area under the curve for each group was calculated and a two-way ANOVA of matched values among time was performed. All these tests were runed by Prism 8^®^ Software (San Diego, CA, USA). Statistical significance was established as *p* < 0.05.

## 3. Results

### 3.1. hCMEC/D3 Infection with NOX5-β Adenovirus Resulted in a Functional Protein Overexpression

**Figure 1:**
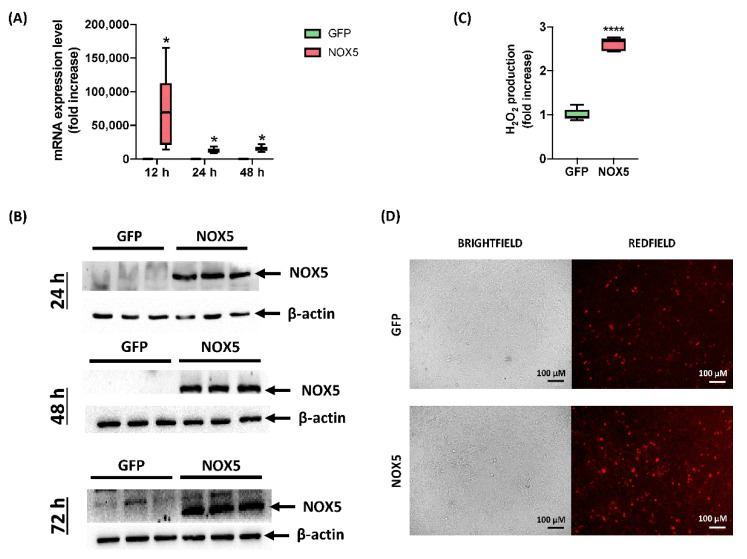
Characterization of hCMEC/D3 infection with GFP and NOX5-β adenoviruses. (**A**) NOX5 mRNA expression level after 12, 24 and 48 h of infection (*n* = 6). (**B**) NOX5 and β-actin immunoblots after 24, 48, and 72 h of infection (*n* = 3). (**C**) Extracellular H_2_O_2_ production after 24 h of infection, measured by AmpliFluRed^TM^ (*n* = 6). (**D**) Superoxide detection by DHE staining after 24 h of infection. Left: brightfield. Right: redfield (oxidized DHE). **GFP:** GFP-infected hCMEC/D3 cells; **NOX5:** NOX5-β-infected hCMEC/D3 cells; * *p* < 0.05 vs. GFP-cells; **** *p* < 0.0001 vs. GFP-cells. Data presented as mean ± SEM.

Although hCMEC/D3 cells infected with GFP adenovirus presented certain NOX5 mRNA levels, when NOX5 adenovirus was employed, it was increased 75,000 times after 12 h of infection. A lighter overexpression continued after 24 and 48 h of infection, accounting approximately for a 10,000-fold increase (Figure 1A). At protein level, NOX5 overexpression was greatly overexpressed 24, 48, and 72 h after infection in NOX5-infected cells (Figure 1B). NOX5 protein was not detected in GFP-infected cells.

In order to test adenoviral NOX5 protein functionality, ROS production was measured by two different techniques. Firstly, AmpliFlu Red^TM^ assay showed that NOX5-infected cells produced almost three times more extracellular H_2_O_2_ than control cells 24 h after infection (Figure 1C). Secondly, DHE staining presented an increased DHE staining as a readout of a higher O_2_^●−^ production (Figure 1D). These data confirmed that hCMEC/D3 cells infected with NOX5-β adenovirus overexpress a functional protein and increase ROS production.

**Figure 2:**
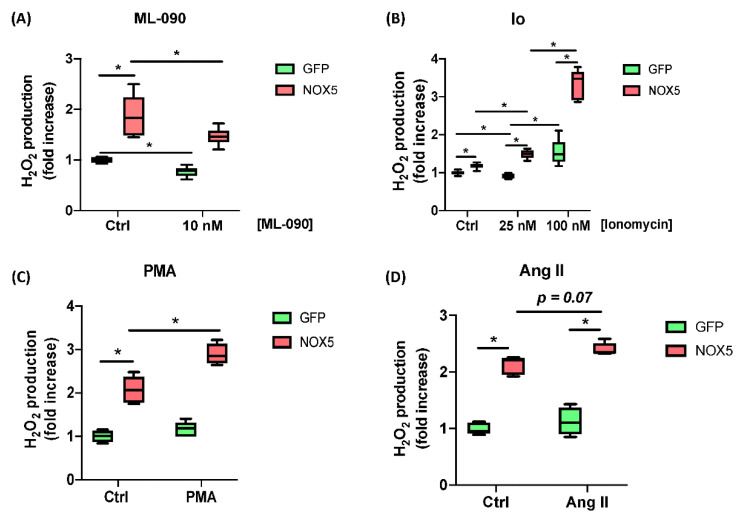
ROS production characterization of hCMEC/D3 cells infected in response to different stimuli and inhibitors. (**A**) H_2_O_2_ production after 10 nM ML-090 inhibition. (**B**) H_2_O_2_ production after 25 and 100 nM Ionomycin (Io) stimulation. (**C**) H_2_O_2_ production after 100 nM PMA stimulation. (**D**) H_2_O_2_ production after 100 nM Ang II stimulation. (*n* = 6). **GFP**: GFP-infected hCMEC/D3 cells; **NOX5:** NOX5-β-infected hCMEC/D3 cells. * *p* < 0.05 vs. indicated group. Data presented as mean ± SEM.

Moreover, the responses of hCMEC/D3-infected cells to ML-090, Ionomycin, PMA, and Ang II were tested. Cells infected with GFP or NOX5-β adenovirus were incubated with 10 nM ML-090, a NOX inhibitor with lower IC50 for NOX5 compared with other family members [26], and extracellular H_2_O_2_ production was measured (Figure 2A). ML-090 significantly inhibited extracellular H_2_O_2_ production in both groups of infected cells at 10 nM. However, the reduction in NOX5-β infected cells lowered almost to control levels. ML-090 reduced in a lesser proportion the ROS production in GFP-infected cells, corroborating the presence of this enzyme at baseline conditions.

Then Ionomycin (Io) was used to increase [Ca^++^] levels and promote NOX5 activity (Figure 2B). In a medium with low Io levels GFP-infected cells did not increase their ROS production (25 nM), while NOX5-infected cells did. At higher Io levels (100 nM) GFP-infected cells were stimulated in a lesser extent that NOX5-infected cells. Finally, PMA (PKC activator) and Ang II were used to stimulate ROS production (Figure 2C,D). Both stimuli produced an increasement in ROS production specific for NOX5 group.

The effect of NOX5-β overexpression in other NOX and antioxidant systems expression was studied (Supplementary Figure S1). NOX1 and NOX2 expression were not detected at mRNA levels, and NOX4 did not suffer any change at any tested time. SOD1, SOD2, and Catalase expression were studied, finding SOD1 downregulation after 24 and 48 h of infection.

### 3.2. NOX5-β Overexpression Induces a Widespread hCMEC/D3 Proteomic Remodelling

**Figure 3:**
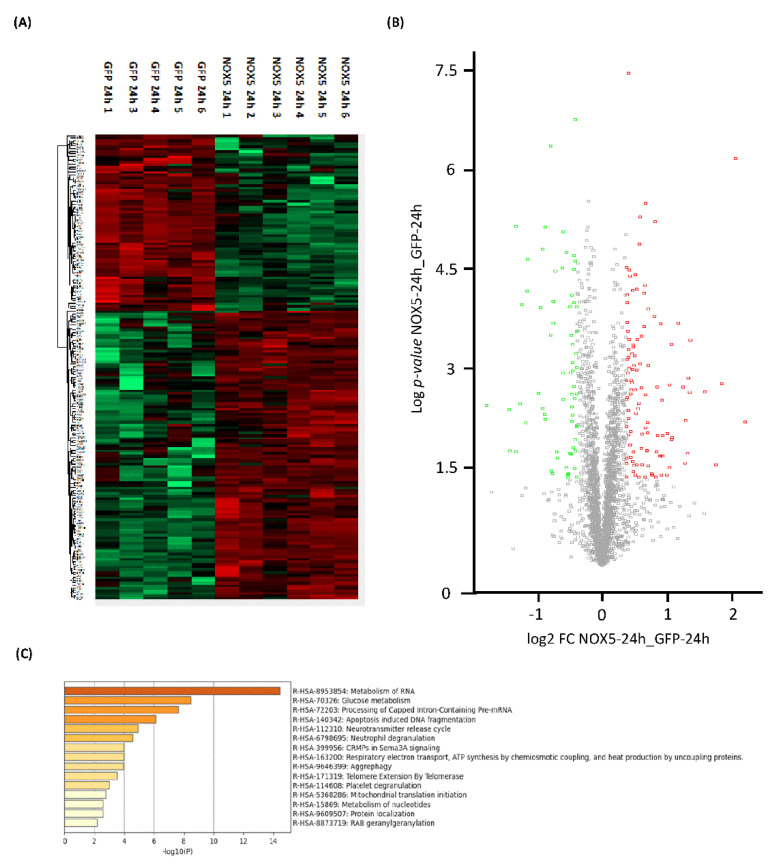
General proteomic analysis of hCMEC/D3 cells infected with GFP and NOX5-β adenovirus for 24 h. (**A**) Heatmap representation of protein differential expression between the two groups. (**B**) Volcano plot representation of samples analyzed. (**C**) Top-15 statistically enriched biofunctions derived from NOX5-β overexpression. **GFP:** GFP-infected hCMEC/D3 cells (*n* = 5); **NOX5:** NOX5-β-infected hCMEC/D3 cells. (*n* = 6).

Proteome heatmap from hCMEC/D3 cells infected with GFP or NOX5-β adenovirus showed marked differences, with 178 significantly dysregulated proteins out of the 2899 analyzed (Figure 3A). A total of 67 proteins were downregulated and 111 were upregulated as indicated by the Volcano Plot (Figure 3B). Among these proteins we found Sideroflexin 3 as the most upregulated one (Fold Change: 4.56), and Histone H2A type-2C as the most downregulated (Fold Change: 0.29).

Using Reactome^®^ software (v79, online), the top 15 significantly dysregulated gene ontology (GO)-terms were identified (Figure 3C). DNA instability processes such as “Apoptotic-induced DNA fragmentation” (R-HSA-140342) and “Telomere Extension by Telomerase” (R-HSA-171319) were found to be altered. Certain processes related with mitochondrial energetics as “Glucose metabolism” (R-HSA-70326), “Respiratory electron transport, ATP synthesis by chemiosmotic coupling, and heat production by uncoupling proteins” (R-HSA-163200) and “Mitochondrial translation initiation” (R-HSA-5368286) were also significantly dysregulated. Moreover, some GO terms related with changes in the expression pattern were found such as: “Metabolism of RNA” (R-HSA-8953854), “Processing of Capped Intron-Containing Pre-mRNA” (R-HSA-72203), “Aggrephagy” (R-HSA-9646399), “Metabolism of nucleotides” (R-HSA-15869), and “Protein localization” (R-HSA-9609507).

**Figure 4:**
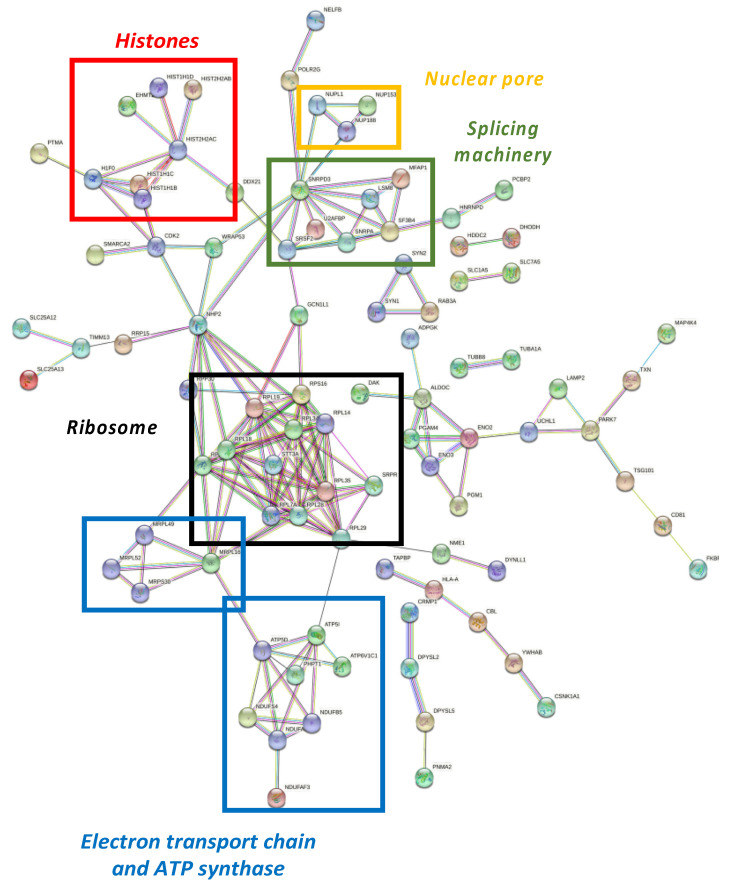
Functional interactome network for differentially expressed proteins in NOX5-β-infected hCMEC/D3. Proteins are presented as nodes and interactions between proteins as connection lines by the STRING^®^ software (v11.5, online). Some protein clusters represent cell structures, which are highlighted by colored squares. These data point out that NOX5-β overexpression alters histones (red), nuclear pores (yellow), splicing machinery (green), ribosomes (black), and the electron transport chain and ATP synthase (blue).

Differential proteome dataset was also introduced in the STRING^®^ metabase to identify different nodes in which these alterations take place. The functional interactome was composed by six subnetworks (Figure 4). Histones, nuclear pores, splicing machinery, ribosomes, the electron transport chain, and ATP-synthase were targeted by NOX5-β overexpression. Among histones (Figure 4, red), the endothelial overexpression of NOX5-β altered different Histone 1 (H1F0, HIST1H1B, HIST1H1C, and HIST1H1D) and Histone 2 (HIST2H2AB and HIST2H2AC) variants, and the Histone 3 epigenetic modulator EHMT2.

Regarding nuclear pore (Figure 4, yellow), the overactivation of NOX5-β dysregulated NUPL1, NUP153, and NUP188 are nucleoporins that conform the nuclear pore appear dysregulated. Around the splicing machinery (Figure 4, green), STRING shows NOX5 effects on SNRPA, SNRPD3, SRSF2, U2AFBP, MFAP1, LSM8, and SF3B4 proteins, all involved in mRNA splicing and maturation. As for the ribosomal network (Figure 4, black), RPL3S, RPL7A, RPL14, RPL18, RPL19, RPL28, RPL29, and RPL34 components from the 60S subunit, and RPS16 from the 40S subunit were dysregulated. Other proteins implicated in protein translation and maturation are present, such as RPP30, involved in tRNA maturation; SRPR, involved in protein translocation to the e.r.; and STT3A, involved in protein N-glycosilation. All these component alterations correlate with GO terms associated with a change in the expression pattern mentioned above.

The STRING^®^ analysis also showed changes associated with cell energetics in NOX5-β overexpressing endothelial cells, affecting electron chain components and promoting ATP synthase alterations (Figure 4, blue). On the one side, NDUFS4, NDUFB5, and NDUFAF3 are components of the electron transport chain, and ATP5D and ATP5I codify for different ATP-synthase subunits. These metabolic components are influenced by PHPT1, a phosphatase that negatively regulates ATP synthesis. On the other side, several alterations in mitochondrial ribosome components (MRPL16, MRPL49, MRPL52, and MRPS30) are present, which affect to the expression of electron transport chain components.

Experimentally demonstrated NOX5 interactors were generated from BIOGRID^®^ (Supplementary Figure S2) and analyzed in the proteomic data obtained (Supplementary Table S1). Among the 46 known interactions, only 14 proteins were detected by the MS/MS technique in this cell type, and among these 14 detected proteins only TUBB8 was altered comparing GFP- and NOX5-β-infected cells. This fact shows that NOX5 overexpression in hCMEC/D3 cells did not induce protein expression changes by protein–protein interactions but by its enzymatic activity as an ROS producer.

### 3.3. Proteomic Analysis of NOX5-β Overexpression in hCMEC/D3 Cells Predicts Alterations in CELL Proliferation and General Metabolism

**Figure 5:**
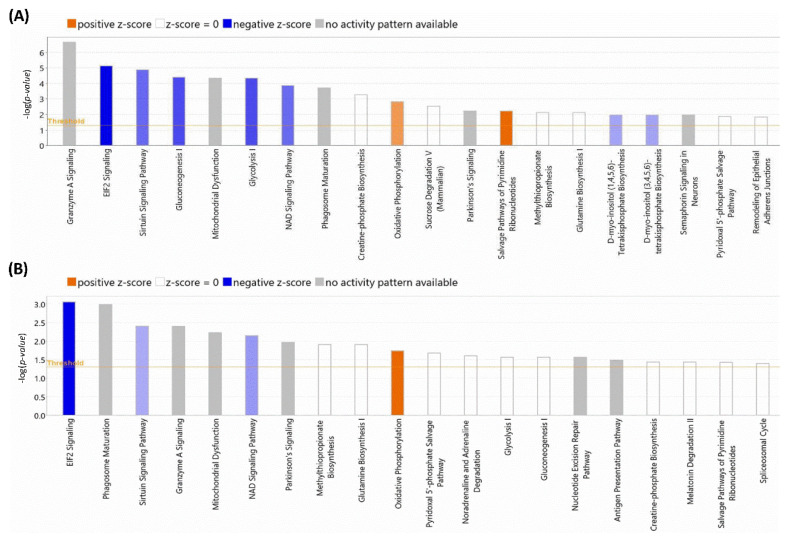
TOP-20 altered cellular processes detected by Ingenuity^®^. (**A**) Processes altered according to the Ingenuity Full Database. (**B**) Processes altered according to the Ingenuity Heart and Cells Database. The size of the bars indicates the log (*p*-value) of each alteration detected. **Blue** colors indicate downregulations and **orange** color indicates upregulations. Color intensity indicates the statistical confidence of each alteration. **Grey** color indicates that it is not possible to analyze the alteration character.

After the initial analysis of NOX5-β overexpression effects in hCMEC/D3 proteome, altered proteins were analyzed by Ingenuity^®^ software (v22.0). This program offered two different results according to the database used. Firstly, we focused on the general analysis, without refining by specific cell types (Figure 5A). NOX5 overexpression significantly altered (with the highest -log(*p*-value)) “Granzyme A Signaling”, a proapoptotic pathway that acts via mitochondria. Moreover, “EIF2 signaling” an inhibitory pathway of global protein translation, was inhibited. Furthermore, NOX5 overexpression downregulated “sirtuin signaling”. The sirtuin signaling pathway is involved in delaying DNA senescence via maintaining its integrity and promoting survival pathways such as MAPK. This fact, together with the inhibition of the “NAD signaling”, an essential component for sirtuins, suggests a pro-senescence role for NOX5.

Also, this general analysis showed alterations in several metabolic pathways. “Gluconeogenesis I” and “Glycolysis I” were significantly downregulated, which indicates that NOX5-β overexpressing cells may have a different glycemic metabolism compared with GFP control cells. This correlates at the mitochondrial level, as “Mitochondrial Dysfunction” is present in the altered pathways. Moreover, an increase in “Oxidative Phosphorylation” is observed when the oxidase is overexpressed. Other metabolic alterations detected are “Phagosome maturation”, “Creatin-phosphate Biosynthesis”, “Sucrose Degradation”, and “Glutamine Biosynthesis I”, which indicates a change in global energetic demand of hCMEC/D3 cells. Finally, the increase in “Salvage Pathway of Pyrimidine Ribonucleotides” is an alternative pathway of ribonucleotide synthesis and a typical feature of tumoral cells. This, together with cell senescence indicates that endothelial cells proliferation capacity can be altered by NOX5, explaining a potentially higher energetic demand. The great majority of these altered pathways were corroborated when specific cardiovascular signaling pathways were used to analyze the data (Figure 5B), supporting the previous statements. 

After this general in silico analysis, the project focused on the study of three different phenotypic alterations: cell proliferation, metabolism, and migration. These alterations were confirmed both by further analysis of the proteomic data, and by specific in vitro studies.

### 3.4. The NOX5-β Overproduction Inhibits Proliferation and Promotes Apoptosis in hCMEC/D3 Cells

Focusing on the proapoptotic and proliferative features observed in the general proteomic analysis, several GO terms related with these characteristics were identified to be altered according to Reactome^®^ software (Supplementary Table S2). 

The first alteration related with this phenotype was genomic instability, cells presented differences in chromatin and telomere maintenance, aberrant transcription and cell senescence, and apoptosis including DNA fragmentation. In addition, general changes in gene expression were detected. NOX5 overexpression affected mRNA biogenesis at different levels including: mRNA maturation and transport between the nucleus and the cytosol; ribosome biogenesis, since rRNA transcription to subunits assembly; tRNA processing; and protein translation and post-translational modifications. Finally, the cell cycle was detected to be altered by NOX5 adenovirus infection. The transition from G2 to mitosis was altered together with different mitotic processes. 

**Figure 6:**
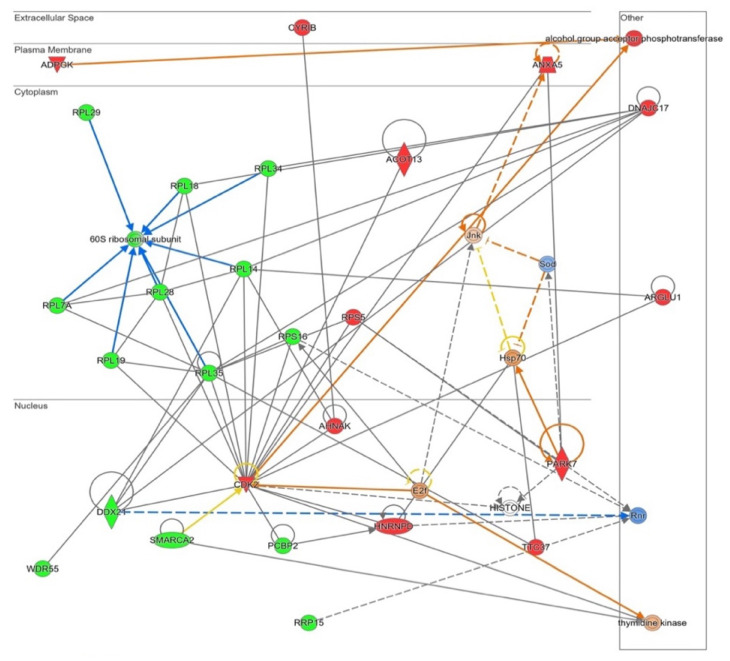
Functional network altered by NOX5-β overexpression in hCMEC/D3 cells related with cell proliferation and apoptosis (inferred from Ingenuity^®^ software). The network contains Cyclins signaling proliferative pathway, including ribosome proteins downregulation. Proteins are represented in different subdivisions of the scheme according to their cellular location (nucleus, cytoplasm, plasma membrane, extracellular space, or other). **Green:** indicates decreased expression measurement. **Red:** indicates increased expression measurement. **Blue lines:** predicted inhibition. **Orange lines:** predicted activation. **Grey/White lines:** non-predicted effect. **Yellow lines:** findings inconsistent with the estate of downstream molecule. Color intensity directly associates with statistical confidence for orange and blue objects. **Potential master regulators identified:** E2f and Hsp70 (activated) and ribonucleotide reductase (Rnr) (inhibited).

These changes related with proliferation and apoptosis had its correlation in an Ingenuity^®^ functional network produced from all altered proteins (Figure 6). The software links several ribosome components’ downregulations (the previously indicated in Figure 4), together with the proposal of E2f as an enhanced potential master regulator of this pathway. A decrease in ribosome presence together with E2f activation may lead to a different level of protein synthesis in NOX5-β-infected cells, which may indicate changes in cell death/proliferation balance.

**Figure 7:**
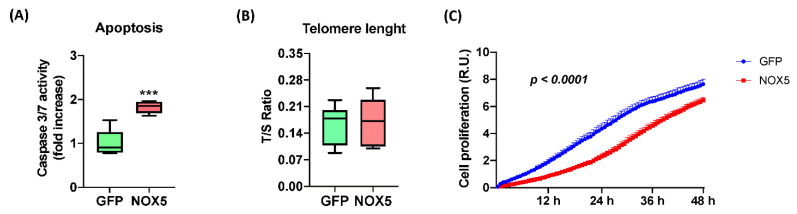
NOX5-β overexpression promotes apoptosis and inhibits cell proliferation in hCMEC/D3 cells. (**A**) Apoptosis measurement by Caspase 3/7 activity. (*n* = 6) (**B**) Telomere length measurement as T/S ratio by qPCR. (*n* = 6) (**C**) Cell proliferation measurement as impedance of E-plate by xCELLigence technology. (*n* = 6–8) **GFP:** GFP-infected hCMEC/D3 cells; **NOX5:** NOX5-β-infected hCMEC/D3 cells. *** *p* < 0.001 vs. GFP-infected cells. Data presented as mean ± SEM. R.U.: relative units. T/S ratio: ratio between telomeres repetitions and single copy gene.

In order to confirm these alterations, several assays were performed to study hCMEC/D3 cell apoptosis and proliferation upon NOX5-β adenovirus infection (Figure 7). NOX5 overexpression promoted apoptosis, measured by Caspase 3/7 activity, reaching almost a 2-fold increase in hCMEC/D3 cells (Figure 7A). This apoptotic increase correlates with several pro-apoptotic proteins deregulated according to proteomic data. Telomere length was predicted to be altered according to Reactome^®^ and Ingenuity^®^; however, qPCR quantification of telomere length did not present differences between the two groups of infected cells (Figure 7B). It is necessary to note that hCMEC/D3 immortalization is achieved by telomerase expression, which can avoid the telomere shortening. Finally, the real time cell proliferation assay showed that NOX5-β-infected cells present significantly lower proliferation rates than GFP cells, at least between 3 h and 48 h of infection (Figure 7C). All these data together show that NOX5 expression in hCMEC/D3 cells promotes apoptosis and inhibits cell proliferation.

### 3.5. NOX5-β Overexpression in hCMEC/D3 Cells Promotes Oxidative Phosphorylation and Energy Expenditure

The general proteomic analysis revealed an effect of NOX5-β overexpression in oxidative phosphorylation and energy production. According to the in silico analysis provided by Reactome^®^, NOX5 protein overexpression produced a general response to cell starvation in hCMEC/D3 cells (Supplementary Table S3). Firstly, carbohydrate energetics were deregulated and glucose metabolism was altered, both at glycolysis and gluconeogenesis levels. Secondly, protein catabolism was activated in response to amino-acid deficiency; this source of energy together with the autophagic process indicates that hCMEC/D3 cells are obtaining extra energy from non-canonical sources. This energy demand correlates at mitochondrial levels. Protein synthesis from mitochondrial DNA is deregulated in NOX5-infected hCMEC/D3 cells. Proteins encoded by mitochondrial translation constitute the electron transport chain, suggesting alterations in these components’ expression. In addition, NOX5 overexpression alters ATP synthesis, affecting electron transport chain and heat production due to ATPase uncoupling.

**Figure 8:**
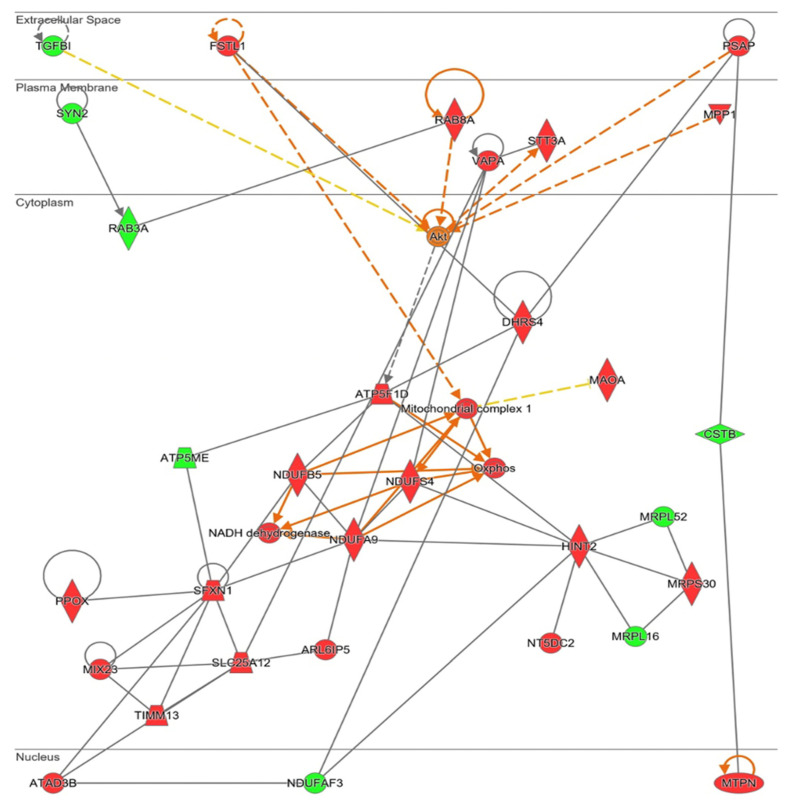
Network altered by NOX5-β overexpression in hCMEC/D3 cells related with metabolic alterations (inferred from Ingenuity^®^ software). Mitochondrial energetics dysregulation, including electron transport chain proteins upregulation. Proteins are represented in different subdivisions of the scheme according to their cellular location (nucleus, cytoplasm, plasma membrane, extracellular space, or other). **Green:** indicates decreased expression measurement. **Red:** indicates increased expression measurement. **Orange lines:** predicted activation. **Grey/White lines:** non-predicted effect. **Yellow lines:** findings inconsistent with the estate of downstream molecule. Color intensity directly associates with statistical confidence for orange and blue objects. **Potential master regulators identified:** Akt (activated).

Ingenuity^®^ software generated a functional network related with mitochondrial energy production and its role in cell homeostasis (Figure 8). In this pathway, mitochondrial ribosome proteins (MRPS30, MRPLS2, and MRPL16) are present, confirming upregulation of mitochondrial protein synthesis. This protein synthesis generated more electron transport chain components, specifically more “Mitochondrial complex I” (MCI, NUDFAF3, NDUFS4, NDUFB5, NDUFA9, ATP5ME, and ATP5F1D) and an increase in oxidative phosphorylation (“Oxphos”). Different mitochondrial transporters that regulate amino acids and proteins transport between mitochondria and cytosol (ARL6IP5, MUX23, SLC25A12, and TIMM13) are upregulated by NOX5-β overexpression. SXFN1 protein, an iron transporter; and PPOX protein, an enzyme implicated in heme group metabolism; are both upregulated, being essential for heme groups of electron transport chain generation. In fact, as stated before, SXFN3 was the most upregulated protein of the analysis. These mitochondrial components’ production requires of ATAD3B a mitochondrial network organizer. The Akt protein is proposed as a master regulator of this pathway by Ingenuity^®^. Finally, some proteins related with cell migration and cell proliferation (DHRS4, FSTL1, MPP1, and TGFBI) are presented too.

**Figure 9:**
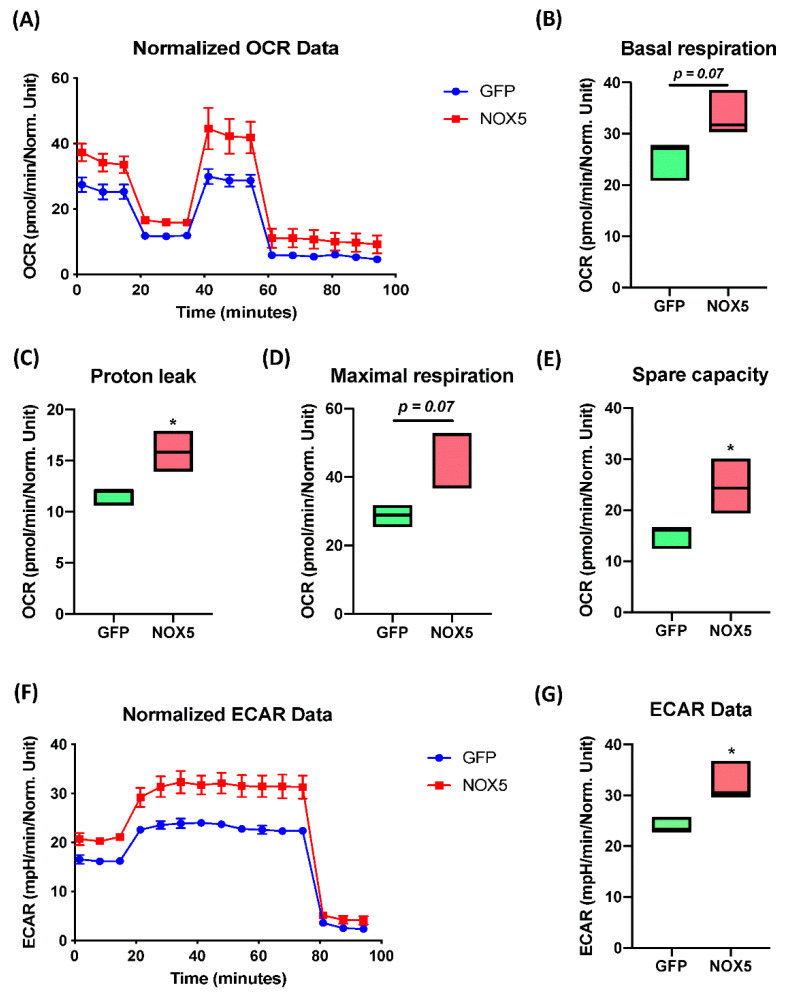
NOX5-β overexpression enhances mitochondrial capacity and activity, as well as non-glucidic metabolism in an Extended Mitostress assay in hCMEC/D3 cells. Data normalized by protein quantity. (**A**) Time course of the oxygen consumption rate (OCR) in GFP and NOX5-infected endothelial cells. Data are presented as: (**B**) cell basal respiration. (**C**) Proton leak. (**D**) Maximal cell respiration. (**E**) Spare capacity. (**F**) Time course of the extracellular acidification rate (ECAR) in GFP and NOX5-infected endothelial cells. (**G**) ECAR data. **OCR:** oxygen consumption rate; **ECAR:** extracellular acidification rate; **GFP:** GFP-infected hCMEC/D3 cells; **NOX5:** NOX5-β-infected hCMEC/D3 cells. (*n* = 3) * *p* < 0.05 vs. GFP-infected cells. Data presented as floating bars from minimum to maximum, marking the median.

The Extended Mitostress assay was able to confirm mitochondrial alterations in hCMEC/D3 cells infected with NOX5-β adenovirus (Figure 9A), showing differences in the time course oxygen consumption rate (OCR) between groups. At basal levels, NOX5-infected cells presented approximately a 50% higher metabolic expense than GFP-infected cells (Figure 9B). However, at baseline, NOX5-infected cells did not present a significantly higher ATP production than GFP-infected cells (data not shown). Considering the proton leak, the assay demonstrates that NOX5 significantly increases proton leak by 50%, reducing the ATP production efficiency in the mitochondria, leading to ATP diffusion to the mitochondrial inner matrix and increasing heat production (Figure 9C). In addition, hCMEC/D3 cells overexpressing NOX5-β are endowed with a higher maximal respiration capacity (Figure 9D). This can serve as a compensatory mechanism to the increased proton leak, together with the upregulation of mitochondrial proteins seen in silico. 

The spare capacity that measures mitochondrial capacity to produce ATP in response to a stress (such as oxidative stress) or an energy demand, appears to be significantly increased in NOX5-infected cells, doubling the capacity of GFP-infected cells (Figure 9E). All these results confirm mitochondrial alterations that were previously predicted by Reactome^®^ and Ingenuity^®^ software. The extended assay allowed us to study non-mitochondrial energy consumption (Figure 9F,G), analyzing general differences in other catabolic pathways. The time course of the extracellular acidification rate (ECAR) data showed a 50% increasement in non-mitochondrial energy consumption, which correlated with the in silico analysis showing alterations in other catabolic pathways. The ECAR results together with the mitochondrial activity analysis confirms alterations in cell energy by NOX5 overexpression.

### 3.6. NOX5-β Overexpression in hCMEC/D3 Cells Promotes Cell Migration and Ezrin/Radixin/Moesin System Dephosphorylation

**Figure 10:**
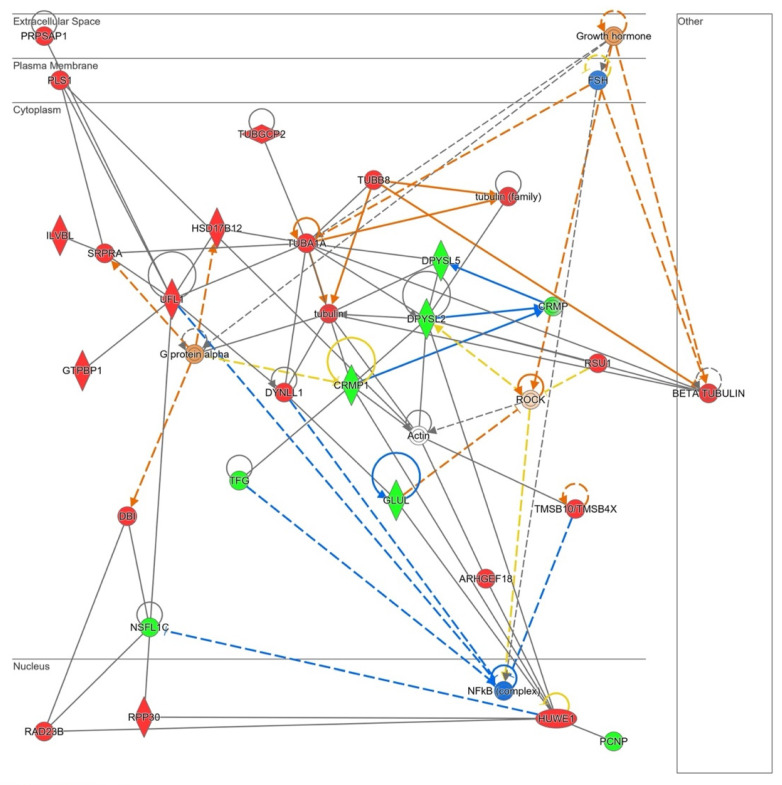
Network altered by NOX5-β overexpression in hCMEC/D3 cells related with cell migration (inferred from Ingenuity^®^ software). Proteins are represented in different subdivisions of the scheme according to their cellular location (nucleus, cytoplasm, plasma membrane, extracellular space, or other). **Green:** indicates decreased expression measurement. **Red:** indicates increased expression measurement. **Blue:** predicted inhibition. **Orange lines:** predicted activation. **Grey/White lines:** non-predicted effect. **Yellow lines:** findings inconsistent with the estate of downstream molecule. Color intensity directly associates with statistical confidence for orange and blue objects. **Potential master regulators identified**: growth hormone and G𝛼-protein (activated), FSH and NF𝜅B (inhibited).

The in silico analysis revealed that NOX5-β overexpression in hCMEC/D3 cells may alter cell migration capacity as it is shown in Supplementary Table S4. Several GO terms that involve cytoskeleton conformational changes were altered by the oxidase. Moreover, the Rho GTPase signaling pathway, which plays a role in cell migration and blood brain barrier permeability, is dysregulated by NOX5 protein. The Ingenuity^®^ analysis also presented a predicted network related with cell migration (Figure 10).

PLS1, a plasma membrane protein that arranges actin filaments appeared upregulated in NOX5-β-infected cells. This protein interacts with several components of the endoplasmic reticulum that participate in protein modifications (ILVBI and NSFL1C) and transport to the Golgi apparatus (NSFL1C, TFG, and SRPRA). UFL1, a protein that participates in endoplasmic reticulum stress, was also upregulated by NOX5 overexpression. This endoplasmic reticulum activity seems to be related with an increase in cytoskeleton proteins production needed for its reorganization. According to Ingenuity^®^ NOX5-infected hCMEC/D3 cells produced more tubulin filaments. TUBA1A, DYNLL1, and TUBB8 which constitute these filaments, are overexpressed. Other proteins that regulate actin filaments configuration, such as PLS1, TUBGCP2, and TMSB1O are dysregulated. ARMGEF18 acts as a regulator of actin fibers formation and cellular shape, and it is related with ROS production levels. In addition, several proteins involved in cell migration as CRMP, CRMP1, and DPYSL2, are altered due to NOX5-β overexpression. GLUL is an enzyme that produces glutamine, essential for endothelial migration. Finally, several master regulators are proposed by Ingenuity^®^, Growth hormone, and FSH that act upstream and regulate cytoskeleton organization; Gα protein that also interacts with the cell skeleton; ROCK, a signaling protein that inhibits inflammatory cell migration and permeability; and NFκB, a transcription factor sensible to ROS levels.

**Figure 11:**
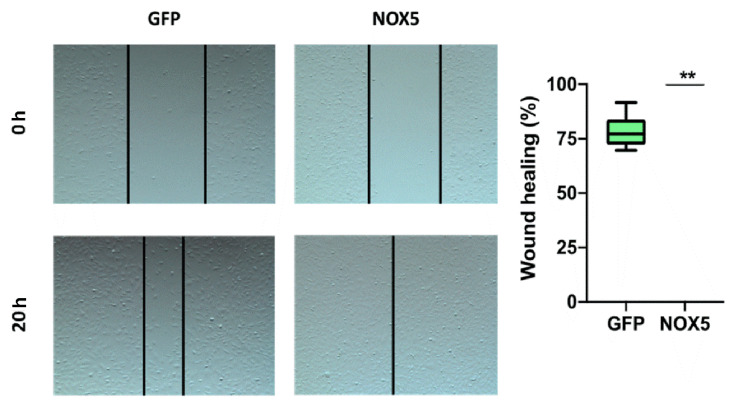
NOX5-β overexpression promotes cell migration in hCMEC/D3 cells. **Left panel:** representative images of wound-healing assay. Pictures were taken 0 and 20 h after the scratch. **Right panel:** wound-healing assay quantification. **GFP:** GFP-infected hCMEC/D3 cells; **NOX5:** NOX5-β-infected hCMEC/D3 cells. (*n* = 6) ** *p* < 0.01 vs. GFP-infected cells. Data presented as mean ± SEM.

To verify the implication of NOX5 in cell migration, a wound-healing assay was performed. After 24 h of cell infection, a wound was generated, and cell migration was analyzed (Figure 11, left panel). hCMEC/D3 cells infected with NOX5-encoding adenovirus totally healed the wound after 20 h. However, hCMEC/D3 cells infected with GFP-encoding adenovirus healed only a 75% of the wound area (Figure 11, right panel). This shows that NOX5 significantly promotes wound healing and cell migration in hCMEC/D3 cells.

**Figure 12:**
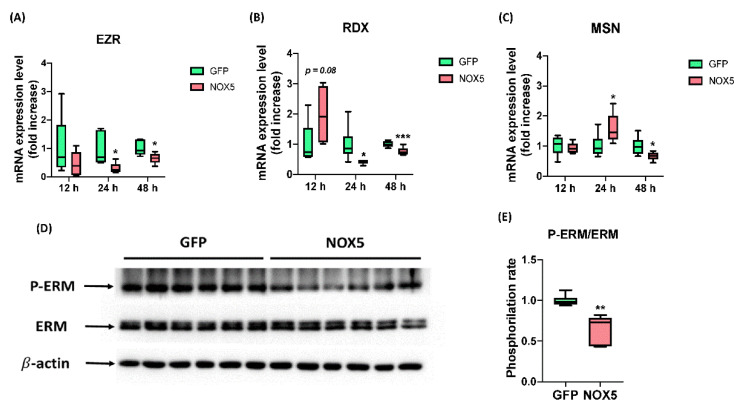
NOX5-β overexpression promotes ERM complex dysregulation and dephosphorylation. (**A**) EZR mRNA expression level after 12, 24, and 48 h of infection (*n* = 6). (**B**) MSN mRNA expression level after 12, 24, and 48 h of infection (*n* = 6). (**C**) RDX mRNA expression level after 12, 24, and 48 h of infection (*n* = 6). (**D**) ERM phosphorylation rate calculated from the previous immunoblot. (**E**) Phosphorylated ERM complex, total ERM complex and β-actin immunoblots after 24 h of infection (*n* = 6). **EZR:** ezrin; **RDX:** radixin; **MSN:** moesin; **ERM:** ezrin-radixin-moesin complex; **GFP:** GFP-infected hCMEC/D3 cells; **NOX5:** NOX5-β-infected hCMEC/D3 cells. * *p* < 0.05 vs. GFP-infected cells, ** *p* < 0.01 vs. GFP-infected cells, *** *p* < 0.001 vs. GFP-infected cells. Data presented as mean ± SEM.

After ensuring that NOX5 promotes migration we studied ERM complex expression and activation, which interacts both with semaphorin and L1 recycling pathway, the promigratory pathways altered in these cells according to Reactome^®^. EZR, MSN, and RDX mRNA levels were studied after 12, 24, and 48 h of infection (Figure 12A–C). No changes in any mRNA expression were found between groups after 12 h of infection. However, EZR and RDX were downregulated after 24 h of infection with NOX5-β adenovirus and remained downregulated but at lower intensity after 48 h. The expression of MSN was increased in NOX5-infected cells after 24 h of infection, and decreased after 48 h hours compared with GFP control cells. Then we studied ERM complex activation by Western blot after 24 h of infection (Figure 12D,E). This experiment showed that ERM complex phosphorylation was significantly reduced and, therefore, more inactive in hCMEC/D3 cells infected with NOX5 adenovirus.

## 4. Discussion

The adenoviral infection of hCMEC/D3 cells with human NOX5-β cDNA generated an acute overexpression of NOX5 that was further stimulated by several ROS triggering stimuli (Ca^++^, PMA, Ang II) and inhibited by ML-090, a NOX-inhibitor with higher affinity to this family member. This NOX5 overexpression model places the potential implications of this oxidase in several processes related to endothelial dysfunction as: (i) cell proliferation and apoptosis, (ii) cell metabolism and mitochondrial dysfunction, and (iii) cell migration. 

Previously, NOX5 activity has been described as a key regulator of vascular smooth muscle cells (VSMCs) phenotypic switching from a synthetic contractile phenotype to a synthetic one in response to Ca^++^ levels. NOX5 silencing decreased synthetic markers expression and increased contractile markers in these cells, while NOX5 overexpression worked in a reverse way [27]. In fact, thrombin dependent NOX5 expression in HAEC leads to actin cytoskeleton changes, monocyte adhesion, and migratory impairment that are prevented with the ROS scavenger N-acetylcysteine [28]. Together with these vascular alterations, NOX5 activity has been related to several CVD such as atherosclerosis [14], stroke post-reoxygenation damage [17], and diabetic nephropathy [18]. Our findings suggest that NOX5 overexpression in endothelial cells can lead to phenotypic changes, cell dysfunction, and thus, be one of the mechanisms that precedes CVD.

The first phenotypic alteration observed was at cell apoptosis and proliferation. NOX5 overexpression enhanced caspase 3/7 levels related with apoptosis, and decreased cell proliferation as it was observed in intact cells by xCELLigence. Our group previously reported that NOX5-β overexpression by this adenovirus in HAECs leads to apoptosis via UPR signaling [19]. Other groups have also described NOX5 as proapoptotic agent. As such, NOX5 stimulation by anlotinib led to tumor cells apoptosis via mitochondrial signaling. NOX5 inhibition reduced anlotinib antitumoral activity, demonstrating its proapoptotic role [29]. This mitochondrial apoptotic pathway can be related to this organelle dysfunction observed in our in vitro model. In addition, NOX5 has been also described as a proliferative oxidase in human hepatic stellate cells LX-2 [30], prostate cancer cells [31], and breast cancer cells [32]. This proliferation enhancement occurs also in two different diabetic nephropathy in vitro models of mesangial cells [33,34]. All together, these data suggest that NOX5 participates in the apoptosis/proliferation balance. In fact, it has been demonstrated in different tumor cell lines, depending on its levels, NOX5 promotes proliferation when it is slightly overexpressed and apoptosis above certain threshold levels [35]. In our in vitro model, the acute and intense NOX5 overexpression overcomes that threshold, so it ultimately leads to cell apoptosis and inhibits proliferation. In this regard, the Ingenuity^®^ software proposed E2f transcription factor as a potential master regulator of the proapoptotic phenotype acquired by hCMEC/D3 cells after NOX5 adenoviral infection. This transcription factor is involved in DNA replication control during the cell cycle. It works as a transition factor through cell cycle phases, the higher the E2f expression, the lower the proliferative stimuli that cells need to replicate [36]. In agreement with this, the reanalysis of a transcriptomic study performed by our group on HAEC infected with NOX5 adenovirus [19], shows that NOX5 promotes the upregulation of different E2f isoforms (Supplementary Table S5). The E2f7 isoform, related with cells apoptosis and DNA damage [36], is the most overexpressed member. These data indicate that NOX5 can activate this transcription factor in endothelial cells, finally leading to apoptosis.

The second phenotypic alteration produced in hCMEC/D3 cells by NOX5 overexpression consisted in metabolic dysregulation. This dysfunction can reinforce the idea of NOX5 promoting apoptosis via mitochondria. NOX5 enhanced cell basal metabolism and altered all the parameters studied in the Mitostress assay: oxidative phosphorylation, ATPase uncoupling, proton leak, spare capacity, and basal metabolism. Previously our group described metabolism implications of endothelial NOX5 in a high fat diet knock in model. These NOX5-expressing mice presented less body weight gain, and less mesenteric and epididymal adipose mass. Moreover, they had lower glycaemia, and improved insulin-induced glucose uptake [37]. In an additional study we found that in these mice NOX5 promoted the expression and activation of specific thermogenesis and lipolysis markers at mesenteric and epididymal fat [38]. Other groups have recently linked NOXes, and more specifically NOX5, with metabolism alterations. For instance, a recent study found that an increase in some diabetes-derived metabolites impairs endothelial nitric oxide synthase and NOX activity in human coronary artery endothelial cells (HCAECs) [39]. In fact, it has been demonstrated in vitro that glucose fluctuations may promote NOX-derived endothelial cell apoptosis in a more intense way than a steady exposure to high glucose levels, which mimics glucose fluctuations of diabetic patients [40]. In human mesangial cells, NOX5 silencing attenuated glucose mediated TXNIP activation, a metabolic protein involved in redox balance that acts via insulin pathway. In a diabetic kidney disease model, the lack of NOX4 and the expression of NOX5 enhanced albuminuria and renal fibrosis were enhanced, in part via TXNIP activation. Finally, NOX5 mesangial expression and TXNIP upregulation correlated in mesangial cells from diabetic patients [41]. Focusing specifically on mitochondrial metabolism, NOXes are related with mitochondrial dysfunction in atrial fibrillation. In fact, apocynin, a NOX inhibitor, reduces mitochondrial dysfunction and its derived oxidative stress, lowering the incidence of atrial fibrillation in diabetic rabbits [42]. Finally, in arterioles from coronary artery disease patients, mitochondrial-derived ROS production was reduced in presence of gp91ds-tat, a NOX inhibitor [43]. These data indicate that NOX-derived ROS can act as upstream regulators of mitochondria and produce its dysfunction in blood vessels.

Finally, hCMEC/D3 infection with NOX5-encoding adenovirus promoted cell migration in the wound-healing assay. Other groups have already related this oxidase with cell migration. On the one hand, NOX5 knock-down prevented in vitro porcine coronary smooth muscle cells migration [44]. On the other hand, NOX5 inhibition in patient-derived VSMCs prevented Ang II-mediated cell migration, suggesting a role of this axis in hypertension [45]. ROS production is also related with this process in endothelial cells. In HAEC, thrombin inhibited cell migration, which was recovered by the ROS scavenger N-acetylcysteine pretreatment [27]. Moreover, NOX4 promoted human umbilical vein cells migration by maintaining VEGF receptor 2 membrane levels [46]. The present results suggest for the first time an implication of NOX5 in endothelial cell migration. Our group studied endothelial ERM complex activation by NOX5 and its involvement in cytoskeletal rearrangements and cell shape. NOX5 overexpression induced changes in mRNA expression levels of ERM components and stimulated complex dephosphorylation. These data suggest that NOX5 can promote endothelial migration via ERM alteration and derived cytoskeletal changes. There is no information of the relationship between NOX5 and this complex, however, it has been described that p47phox and p40phox protein subunits of NOX2 can interact with the moesin component [47]. Moreover, an ERM interactor protein, EBP50 (ERM binding protein 50), regulates p47phox binding to NOX1 in murine VSMCs [48]. These works demonstrate a regulatory relationship between ERM complex and NOX activity. Finally, it was described that Ang II mediates VSMCs migration by ROS activation of ERM signaling among other pathways [49], which suggests a crosstalk between Ang II (a NOX5 stimulator), ROS production, ERM complex, and cell migration.

These three features (cell apoptosis, mitochondrial dysfunction, and cell migration) are present in CVDs such as atherosclerosis, myocardial infarction, and stroke. Firstly, endothelial cell death after an oxidative stress situation produces extracellular vesicles that favor endothelium erosion, thrombosis, and vessel occlusion [50]. Secondly, ROS overproduction via mitochondrial dysfunction leads to inflammation in endothelial cells and promotes development of atherosclerotic plaque [51]. In agreement with this idea, mitochondria have been proposed as potential therapeutic targets in stroke, as mitochondrial dysfunction promotes cell death among other alterations in this disease [52]. Finally, endothelial cell cytoskeleton changes may increase cell permeability; for example, endothelial cells stimulated with curcumin are more permeable to monocyte migration [53]. Several groups have demonstrated how ERM complex and Rho GTPase signaling can mediate the endothelial infiltration of certain types of immune cells [54,55,56].

## 5. Conclusions

In conclusion, NOX5 overexpression may favor endothelial dysfunction and participate in the onset of CVDs such as atherothrombosis or stroke by promoting apoptosis, mitochondrial dysfunction, and cytoskeleton changes. Nevertheless, our proteomic approach should lead to future studies about endothelial NOX5 overexpression effects in vivo to identify the exact role of these phenotypic alterations in disease models. Therefore, NOX5 targeting can emerge as a novel therapeutic strategy for the prevention of CVDs. In this way, this work can serve as a start point for additional works to study the signaling pathways in which NOX5 produces its detrimental effects, thus increasing the knowledge of this potential therapeutic strategy.

## Figures and Tables

**Table 1:** Primers used for specific gene expression quantification by qPCR.

Gene	Accession nº	Primer	Sequence (5′-3′)
NOX1	NM_007052.5	Forward	CTACCTCCCACCCCAAGTCT
		Reverse	TGACTGCTCAAACCTGACGA
NOX2	NM_000433.4	Forward	CTGTGAATGAGGGGCTCTCC
		Reverse	GCAATGGTGTGAATCGCAGA
NOX4	NM_016931.5	Forward	CTGTATTTTCTCAGGCGTGCAT
		Reverse	CCTCATCTCGGTATCTTGCTGC
NOX5	NM_024505.4	Forward	TAAGAGGCTGTCGAGGAGTGT
		Reverse	CCAAAAGTATCTCAGAGCCCTTG
SOD1	NM_000454.5	Forward	GAAGAGAGGCATGTTGGAGAC
		Reverse	GAATGTTTATTGGGCGATCC
SOD2	NM_000636.4	Forward	GTTGGCCAAGGGAGATGTT
		Reverse	TCAAAGGAACCAAAGTCACG
Catalase	NM_001752.4	Forward	CTCCACTGTTGCTGGAGAATC
		Reverse	AGAAGTCCCAGACCATGTCC
EZR	NM_003379.5	Forward	CTGGAGCGGCAACAGCTG
		Reverse	CTGTCTCTCCAGCTCCTCCT
MSN	NM_002444.3	Forward	CGCAGAGAGTCCTGGAACA
		Reverse	TTCACTCCAGGGGGAAGCCTA
RDX	NM_001260492.2	Forward	CGCTCTCCGGAAAGTGATAACA
		Reverse	AGACCTCACGCAAACCAACT
E-selectin	NM_000450.2	Forward	CTGCTTCCCAAAACGGAAAGT
		Reverse	GCTTCCGTGGAGGTGTTGTA
GAPDH	NM_001289726.1	Forward	ATGACAACTTTGTCAAGCTCATTT
		Reverse	GGTCCACCACCCTGTTGCT

**NOX1:** NADPH oxidase 1; **NOX2:** NADPH oxidase 2; **NOX4:** NADPH oxidase 4; **NOX5:** NADPH oxidase 5; **SOD1:** superoxide dismutase 1; **SOD2:** superoxide dismutase 2; **EZR:** ezrin; **MSN:** moesin; **RDX:** radixin; **GAPDH:** glyceraldehyde 3-phosphate dehydrogenase.

## Data Availability

MS data and search results files were deposited in the ProteomeXchange Consortium via the JPOST partner repository (https://repository.jpostdb.org, accessed on 25 October 2022) [23] with the identifier PXD034610 for ProteomeXchange and JPST001668 https://repository.jpostdb.org/entry/JPST001668.0, accessed on 25 October 2022, for jPOST (for reviewers: https://repository.jpostdb.org/preview/126903203362ab3b089787b, accessed on 25 October 2022; Access key: 5498).

## Supplementary Materials

The following supporting information can be downloaded at: https://www.mdpi.com/article/10.3390/antiox11112147/s1, Figure S1: Analysis of different antioxidant systems and other NADPH oxidases at mRNA levels; Figure S2: Human NOX5 interactome analysis from Biogrid metabase; Table S1: Analysis of NOX5 protein interactors from Biogrid metabase in hCMEC/D3 cells infected with GFP or NOX5-β encoding adenovirus; Table S2: GO terms related with proliferative and apoptotic processes altered in NOX5-β-infected hCMEC/D3 cells according to Reactome^®^ software; Table S3: GO terms related with metabolic alterations in NOX5-β-infected hCMEC/D3 cells according to Reactome^®^ software; Table S4: GO terms related with cell migration in NOX5-𝛽-infected hCMEC/D3 cells according to Reactome^®^ software; Table S5: E2f transcription factor isoforms expression in NOX5-β-infected HAEC transcriptomic array after 12, 18 and 24 h of infection; Table S6: Rho GTPase family members expression in NOX5-β-infected HAEC transcriptomic array after 12, 18, and 24 h of infection.

## References

[1] Godo S., Shimokawa H. (2017). Endothelial Functions. Arter. Thromb. Vasc. Biol..

[2] Furchgott R.F., Zawadzki J.V. (1980). The obligatory role of endothelial cells in the relaxation of arterial smooth muscle by acetylcholine. Nature.

[3] Hong N., Ye Z., Lin Y., Liu W., Xu N., Wang Y. (2021). Agomelatine prevents angiotensin II-induced endothelial and mononuclear cell adhesion. Aging.

[4] Guillot N., Kollins D., Gilbert V., Xavier S., Chen J., Gentle M., Reddy A., Bottinger E., Jiang R., Rastaldi M.P. (2012). BAMBI Regulates Angiogenesis and Endothelial Homeostasis through Modulation of Alternative TGFβ Signaling. PLoS ONE.

[5] Daiber A., Steven S., Weber A., Shuvaev V.V., Muzykantov V.R., Laher I., Li H., Lamas S., Münzel T. (2017). Targeting vascular (endothelial) dysfunction. Br. J. Pharmacol..

[6] Li H., Horke S., Förstermann U. (2014). Vascular oxidative stress, nitric oxide and atherosclerosis. Atherosclerosis.

[7] Lozhkin A., Vendrov A.E., Pan H., Wickline S.A., Madamanchi N.R., Runge M.S. (2017). NADPH oxidase 4 regulates vascular inflammation in aging and atherosclerosis. J. Mol. Cell Cardiol..

[8] Yu W., Xiao L., Que Y., Li S., Chen L., Hu P., Xiong R., Seta F., Chen H., Tong X. (2020). Smooth muscle NADPH oxidase 4 promotes angiotensin II-induced aortic aneurysm and atherosclerosis by regulating osteopontin. Biochim. Biophys. Acta Mol. Basis Dis..

[9] Ngarashi D., Fujikawa K., Ferdaus M.Z., Zahid H.M., Ohara H., Nabika T. (2019). Dual inhibition of NADPH oxidases and xanthine oxidase potently prevents salt-induced stroke in stroke-prone spontaneously hypertensive rats. Hypertens. Res..

[10] Vara D., Mailer R.K., Tarafdar A., Wolska N., Heestermans M., Konrath S., Spaeth M., Renné T., Schröder K., Pula G. (2021). NADPH Oxidases Are Required for Full Platelet Activation In Vitro and Thrombosis In Vivo but Dispensable for Plasma Coagulation and Hemostasis. Arter. Thromb. Vasc. Biol..

[11] Marqués J., Cortés A., Pejenaute Á., Zalba G. (2020). Implications of NADPH oxidase 5 in vascular diseases. Int. J. Biochem. Cell Biol..

[12] Da Silva J.F., Alves J.V., Silva-Neto J.A., Costa R.M., Neves K.B., Alves-Lopes R., Carmargo L.L., Rios F.J., Montezano A.C., Touyz R.M. (2021). Lysophosphatidylcholine induces oxidative stress in human endothelial cells via NOX5 activation—Implications in atherosclerosis. Clin. Sci..

[13] Marqués J., Cortés A., Pejenaute Á., Ansorena E., Abizanda G., Prósper F., Martínez-Irujo J.J., de Miguel C., Zalba G. (2020). Induction of Cyclooxygenase-2 by Overexpression of the Human NADPH Oxidase 5 (NOX5) Gene in Aortic Endothelial Cells. Cells.

[14] Guzik T.J., Chen W., Gongora M.C., Guzik B., Lob H.E., Mangalat D., Hoch N., Dikalov S., Rudzinski P., Kapelak B. (2008). Calcium-Dependent NOX5 Nicotinamide Adenine Dinucleotide Phosphate Oxidase Contributes to Vascular Oxidative Stress in Human Coronary Artery Disease. J. Am. Coll. Cardiol..

[15] Petheő G.L., Kerekes A., Mihálffy M., Donkó Á., Bodrogi L., Skoda G., Baráth M., Hoffmann O.I., Szeles Z., Balázs B. (2021). Disruption of the NOX5 Gene Aggravates Atherosclerosis in Rabbits. Circ. Res..

[16] Cortés A., Solas M., Pejenaute Á., Abellanas M.A., Garcia-Lacarte M., Aymerich M.S., Marqués J., Ramírez M.J., Zalba G. (2021). Expression of Endothelial NOX5 Alters the Integrity of the Blood-Brain Barrier and Causes Loss of Memory in Aging Mice. Antioxidants.

[17] Casas A.I., Kleikers P.W., Geuss E., Langhauser F., Adler T., Busch D.H., Gailus-Durner V., De Angelis M.H., Egea J., Lopez M.G. (2019). Calcium-dependent blood-brain barrier breakdown by NOX5 limits postreperfusion benefit in stroke. J. Clin. Investig..

[18] Deliyanti D., AlRashdi S.F., Touyz R.M., Kennedy C.R., Jha J.C., Cooper M.E., Jandeleit-Dahm K.A., Wilkinson-Berka J.L. (2020). Nox (NADPH Oxidase) 1, Nox4, and Nox5 Promote Vascular Permeability and Neovascularization in Retinopathy. Hypertension.

[19] Cortés A., Pejenaute Á., Marqués J., Izal Í., Cenoz S., Ansorena E., Martínez-Irujo J.J., de Miguel C., Zalba G. (2021). NADPH Oxidase 5 Induces Changes in the Unfolded Protein Response in Human Aortic Endothelial Cells and in Endothelial-Specific Knock-in Mice. Antioxidants.

[20] Shevchenko A., Tomas H., Havli J., Olsen J.V., Mann M. (2006). In-gel digestion for mass spectrometric characterization of proteins and proteomes. Nat. Protoc..

[21] Cox J., Mann M. (2008). MaxQuant enables high peptide identification rates, individualized p.p.b.-range mass accuracies and proteome-wide protein quantification. Nat. Biotechnol..

[22] Cox J., Neuhauser N., Michalski A., Scheltema R.A., Olsen J.V., Mann M. (2011). Andromeda: A Peptide Search Engine Integrated into the MaxQuant Environment. J. Proteome Res..

[23] Tyanova S., Temu T., Sinitcyn P., Carlson A., Hein M.Y., Geiger T., Mann M., Cox J. (2016). The Perseus computational platform for comprehensive analysis of (prote)omics data. Nat. Methods.

[24] Okuda S., Watanabe Y., Moriya Y., Kawano S., Yamamoto T., Matsumoto M., Takami T., Kobayashi D., Araki N., Yoshizawa A.C. (2017). jPOSTrepo: An international standard data repository for proteomes. Nucleic Acids Res..

[25] Pejenaute Á., Cortés A., Marqués J., Montero L., Beloqui Ó., Fortuño A., Martí A., Orbe J., Zalba G. (2020). NADPH Oxidase Overactivity Underlies Telomere Shortening in Human Atherosclerosis. Int. J. Mol. Sci..

[26] Dao V.T., Elbatreek M.H., Altenhöfer S., Casas A.I., Machado M.P., Neullens C.T., Knaus U.G., Schmidt H.H.H.W. (2020). Isoform-selective NADPH oxidase inhibitor panel for pharmacological target validation. Free Radic. Biol. Med..

[27] Furmanik M., Chatrou M., Van Gorp R., Akbulut A., Willems B., Schmidt H., Van Eys G., Bochaton-Piallat M.-L., Proudfoot D., Biessen E. (2020). Reactive Oxygen-Forming Nox5 Links Vascular Smooth Muscle Cell Phenotypic Switching and Extracellular Vesicle-Mediated Vascular Calcification. Circ. Res..

[28] Pai W.-Y., Lo W.-Y., Hsu T., Peng C.-T., Wang H.-J. (2017). Angiotensin-(1–7) Inhibits Thrombin-Induced Endothelial Phenotypic Changes and Reactive Oxygen Species Production via NADPH Oxidase 5 Downregulation. Front. Physiol..

[29] Huang Z., Su Q., Li W., Ren H., Huang H., Wang A. (2021). Suppressed mitochondrial respiration via NOX5-mediated redox imbalance contributes to the antitumor activity of anlotinib in oral squamous cell carcinoma. J. Genet. Genom..

[30] Andueza A., Garde N., García-Garzón A., Ansorena E., López-Zabalza M.J., Iraburu M.J., Zalba G., Martínez-Irujo J.J. (2018). NADPH oxidase 5 promotes proliferation and fibrosis in human hepatic stellate cells. Free Radic. Biol. Med..

[31] Brar S.S., Corbin Z., Kennedy T.P., Hemendinger R., Thornton L., Bommarius B., Arnold R.S., Whorton A.R., Sturrock A.B., Huecksteadt T.P. (2003). NOX5 NAD(P)H oxidase regulates growth and apoptosis in DU 145 prostate cancer cells. Am. J. Physiol. Cell Physiol..

[32] Dho S.H., Kim J.Y., Lee K.-P., Kwon E.-S., Lim J.C., Kim C.-J., Jeong D., Kwon K.-S. (2017). STAT5A-mediated NOX5-L expression promotes the proliferation and metastasis of breast cancer cells. Exp. Cell Res..

[33] Wu J., Lu K., Zhu M., Xie X., Ding Y., Shao X., Chen Y., Liu J., Xu M., Xu Y. (2020). miR-485 suppresses inflammation and proliferation of mesangial cells in an in vitro model of diabetic nephropathy by targeting NOX5. Biochem. Biophys. Res. Commun..

[34] Li Y., Li Y., Zheng S. (2020). Inhibition of NADPH Oxidase 5 (NOX5) Suppresses High Glucose-Induced Oxidative Stress, Inflammation and Extracellular Matrix Accumulation in Human Glomerular Mesangial Cells. Med. Sci. Monit..

[35] Dho S.H., Kim J.Y., Kwon E.-S., Lim J.C., Park S.S., Kwon K.-S. (2015). NOX5-L can stimulate proliferation and apoptosis depending on its levels and cellular context, determining cancer cell susceptibility to cisplatin. Oncotarget.

[36] Kent L.N., Leone G. (2019). The broken cycle: E2F dysfunction in cancer. Nat. Rev. Cancer.

[37] García J.G., Ansorena E., Milagro F.I., Zalba G., de Miguel C. (2021). Endothelial Nox5 Expression Modulates Glucose Uptake and Lipid Accumulation in Mice Fed a High-Fat Diet and 3T3-L1 Adipocytes Treated with Glucose and Palmitic Acid. Int. J. Mol. Sci..

[38] García J.G., de Miguel C., Milagro F.I., Zalba G., Ansorena E. (2021). Endothelial NOX5 Expression Modulates Thermogenesis and Lipolysis in Mice Fed with a High-Fat Diet and 3T3-L1 Adipocytes through an Interleukin-6 Dependent Mechanism. Antioxidants.

[39] Ren X., Ren L., Wei Q., Shao H., Chen L., Liu N. (2017). Advanced glycation end-products decreases expression of endothelial nitric oxide synthase through oxidative stress in human coronary artery endothelial cells. Cardiovasc. Diabetol..

[40] Quagliaro L., Piconi L., Assaloni R., Martinelli L., Motz E., Ceriello A. (2003). Intermittent High Glucose Enhances Apoptosis Related to Oxidative Stress in Human Umbilical Vein Endothelial Cells: The role of protein kinase C and NAD(P)H-oxidase activation. Diabetes.

[41] Jha J.C., Dai A., Garzarella J., Charlton A., Urner S., Østergaard J.A., Okabe J., Holterman C.E., Skene A., Power D.A. (2022). Independent of Renox, NOX5 Promotes Renal Inflammation and Fibrosis in Diabetes by Activating ROS-Sensitive Pathways. Diabetes.

[42] Zhou L., Liu Y., Wang Z., Liu D., Xie B., Zhang Y., Yuan M., Tse G., Li G., Xu G. (2021). Activation of NADPH oxidase mediates mitochondrial oxidative stress and atrial remodeling in diabetic rabbits. Life Sci..

[43] Zinkevich N.S., Fancher I.S., Gutterman D.D., Phillips S.A. (2017). Roles of NADPH oxidase and mitochondria in flow-induced vasodilation of human adipose arterioles: ROS-induced ROS release in coronary artery disease. Microcirculation.

[44] Gole H.K.A., Tharp D.L., Bowles D.K. (2014). Upregulation of Intermediate-Conductance Ca2+-Activated K+ Channels (KCNN4) in Porcine Coronary Smooth Muscle Requires NADPH Oxidase 5 (NOX5). PLoS ONE.

[45] Camargo L.L., Montezano A.C., Hussain M., Wang Y., Zou Z., Rios F.J., Neves K.B., Alves-Lopez R., Awan F.R., Guzik T.J. (2022). Central role of c-Src in NOX5- mediated redox signalling in vascular smooth muscle cells in human hypertension. Cardiovasc. Res..

[46] Miyano K., Okamoto S., Yamauchi A., Kawai C., Kajikawa M., Kiyohara T., Tamura M., Taura M., Kuribayashi F. (2020). The NADPH oxidase NOX4 promotes the directed migration of endothelial cells by stabilizing vascular endothelial growth factor receptor 2 protein. J. Biol. Chem..

[47] Wientjes F.B., Reeves E.P., Soskic V., Furthmayr H., Segal A.W. (2001). The NADPH Oxidase Components p47phox and p40phox Bind to Moesin through Their PX Domain. Biochem. Biophys. Res. Commun..

[48] Ghouleh I.A., Meijles D.N., Mutchler S., Zhang Q., Sahoo S., Gorelova A., Amaral J.H., Rodríguez A.I., Mamonova T., Song G.J. (2016). Binding of EBP50 to Nox organizing subunit p47 phox is pivotal to cellular reactive species generation and altered vascular phenotype. Proc. Natl. Acad. Sci. USA.

[49] Zhang Y.-X., Xu J.-T., You X.-C., Wang C., Zhou K.-W., Li P., Sun P., Wang L., Wang T.-H. (2016). Inhibitory Effects of Hydrogen on Proliferation and Migration of Vascular Smooth Muscle Cells via Down-Regulation of Mitogen/Activated Protein Kinase and Ezrin/Radixin/Moesin Signaling Pathways. Chin. J. Physiol..

[50] Paone S., Baxter A.A., Hulett M.D., Poon I.K.H. (2019). Endothelial cell apoptosis and the role of endothelial cell-derived extracellular vesicles in the progression of atherosclerosis. Cell Mol. Life Sci..

[51] Li Z., Li Q., Wang L., Li C., Xu M., Duan Y., Ma L., Li T., Chen Q., Wang Y. (2021). Targeting mitochondria-inflammation circle by renal denervation reduces atheroprone endothelial phenotypes and atherosclerosis. Redox Biol..

[52] He Z., Ning N., Zhou Q., Khoshnam S.E., Farzaneh M. (2020). Mitochondria as a therapeutic target for ischemic stroke. Free Radic. Biol. Med..

[53] Monfoulet L.-E., Mercier S., Bayle D., Tamaian R., Barber-Chamoux N., Morand C., Milenkovic D. (2017). Curcumin modulates endothelial permeability and monocyte transendothelial migration by affecting endothelial cell dynamics. Free Radic. Biol. Med..

[54] Yamamoto K., Takagi Y., Ando K., Fukuhara S. (2021). Rap1 Small GTPase Regulates Vascular Endothelial-Cadherin-Mediated Endothelial Cell–Cell Junctions and Vascular Permeability. Biol. Pharm. Bull..

[55] Newe A., Rzeniewicz K., König M., Schroer C.F.E., Joachim J., Rey-Gallardo A., Marrink S.J., Deka J., Parsons M., Ivetic A. (2019). Serine Phosphorylation of L-Selectin Regulates ERM Binding, Clustering, and Monocyte Protrusion in Transendothelial Migration. Front. Immunol..

[56] Rey-Gallardo A., Tomlins H., Joachim J., Rahman I., Kitscha P., Frudd K., Parsons M., Ivetic A. (2018). Sequential binding of Ezrin and Moesin to L-selectin regulates monocyte protrusive behaviour during transendothelial migration. J. Cell Sci..

