# Supplementary material for: NADPH Oxidase 5 (NOX5) Overexpression Promotes Endothelial Dysfunction via Cell Apoptosis, Migration, and Metabolic Alterations in Human Brain Microvascular Endothelial Cells (hCMEC/D3)
Source: Antioxidants (Basel). 2022 Oct 29;11(11):2147. doi: 10.3390/antiox11112147 (PMC9686921; doi:10.3390/antiox11112147)
Supplement: Supplementary file 1 [file antioxidants-11-02147-s001.zip › antioxidants-1993861-Supplementary.pdf]

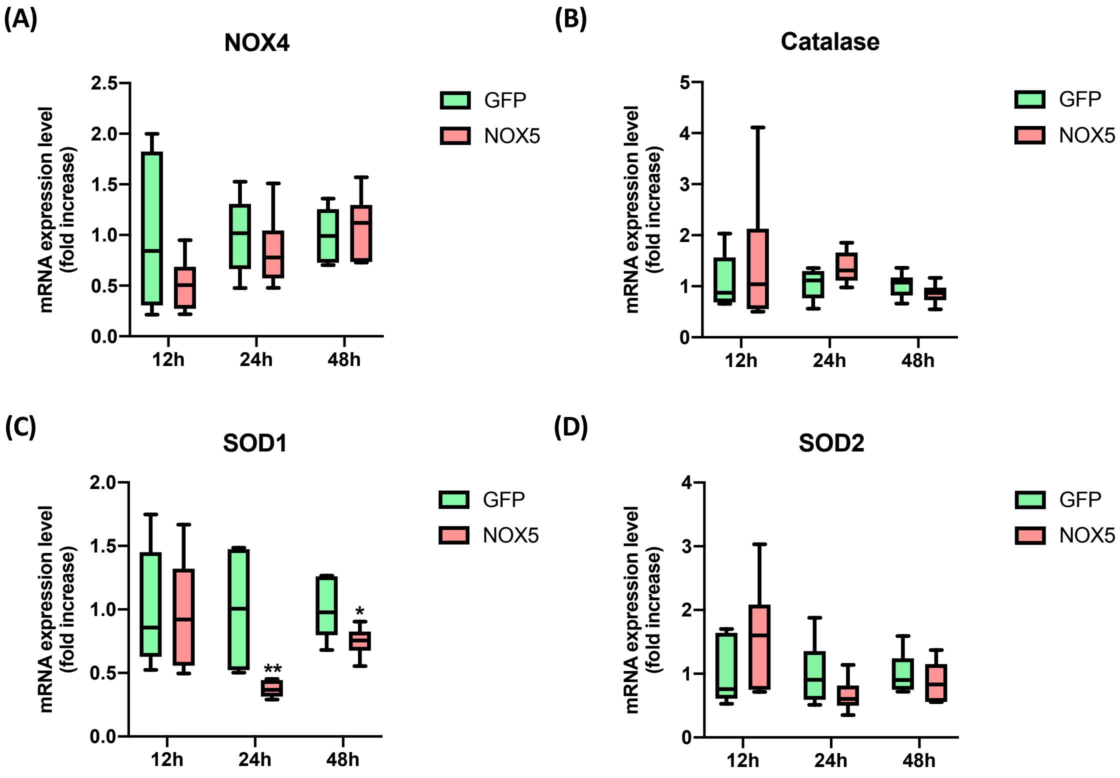

Supplementary Figure S1. Analysis of different antioxidant systems and other NADPH oxidases at mRNA levels. (A): NOX4 expression. **(B):** Catalase expression. **(C):** SOD1 expression. **(D):** SOD2 expression. NOX1 and NOX2 were not detected by qPCR in any group. GFP-infected hCMEC/D3 cells. **NOX5:** NOX5-β-infected hCMEC/ D3 cells. **NOX4:** NADPH oxidase 4. **SOD1:** superoxide dismutase 1. **SOD2:** superoxide dismutase 2. (*n=6*), (*) $p<0.05$ vs GFP-cells, (**) $p<0.01$ vs GFP-cells. Data presented as mean±SEM.

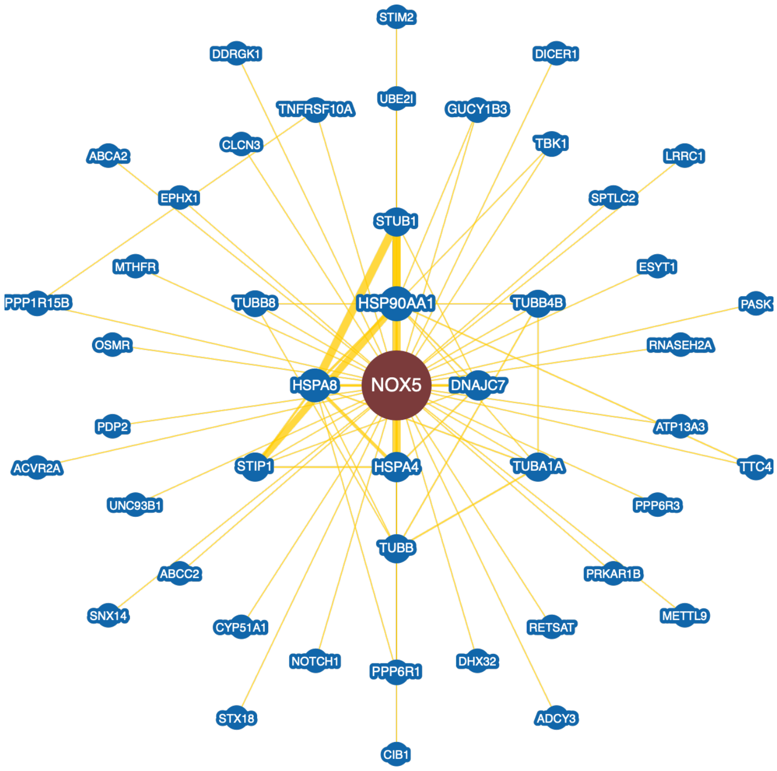

Supplementary Figure S2. Human NOX5 interactome analysis from Biogrid metabase. ABCA2: ATP-binding cassette sub-family A member 2. **ABCC2:** ATP-binding cassette sub-family C member 2. **ACVR2A:** activin A type IIA receptor. **ADCY3:** adenylate cyclase 3. **ATP13A3:** ATPase type 13A3. **CIB1:** calcium and integrin binding protein 1. **CLCN3:** chloride channel 3. **CYP51A1:** cytochrome P450. **DDRGK1:** DDRGK domain containing protein 1. **DHX32:** DEAH box polypeptide 32. **DICER1:** dicer 1 (ribonuclease type 3). **DNAJC7:** DnaJ (Hsp40) homolog member 7. **EPHX1:** epoxide hydrolase 1. **ESYT1:** extended synaptotagmin-like protein 1. **GUCY1B3:** Guanylate cyclase 1-β3. **HSP90AA1:** Heat shock 90 kDa protein α-A1. **HSPA4:** heat shock 70 kDa protein 4. **HSPA8:** heat shock 70 kDa protein 8. **LRRC1:** leucine rich repeat containing protein 1. **METTL9:** methyltransferase like protein 9. **MTHFR:** methylenetetrahydrofolate reductase. **NOTCH1:** notch receptor 1. **OSMR:** oncostatin M receptor. **PASK:** PAS domain containing serin/threonine kinase. **PDP2:** pyruvate dehyrogenase phosphatase catalytic subunit 2. **PPP1R15B:** protein phosphatase 1 regulatory subunit 15B. **PPP6R1:** protein phosphatase 6 regulatory subunit 1. **PPP6R3:** protein phosphatase 6 regulatory subunit 3. **PRKAR1B:** protein kinase cAMP-dependent type I-β. **RETSAT:** retinol saturase. **RNASEH2A:** ribonuclease H2 subunit A. **SNX14:** sorting nexin 14. **SPTLC2:** serine palmitoyltransferase long chain base subunit 2. **STIM2:** stromal interaction molecule 2. **STIP1:** stress-induced phosphoprotein 1. **STUB1:** E3 ubiquitin protein ligase. **STX18:** syntaxin 18. **TBK1:** TANK-binding kinase 1. **TNFRSF10A:** tumor necrosis factor receptor superfamily member 10a. **TUBA1A:** tubulin α-1A. **TUBB:** tubulin β-1. **TUBB4B:** tubulin β-4B. **TTC4:** tetratricopeptide repeat domain 4. **UBE2I:** ubiquitin-conjugating enzyme E2I. **UNC93B1:** unc-93 homolog B1.

Supplementary Table S1. Analysis of NOX5 protein interactors from BIOGRID® software in hCMEC/D3 cells infected with GFP or NOX5-β encoding adenovirus. Q: quantified. NQ: not quantified. A: altered. NA: not altered.

Protein	Uniprot code	Quantified	Altered
ABCA2	Q9BZC7	NQ	-
ABCC2	Q92887	NQ	-
ACVR2A	P27037	NQ	-
ADCY3	O60266	NQ	-
ATP13A3	Q9H7F0	NQ	-
CIB1	Q99828	NQ	-
CLCN3	P51790	NQ	-
CYP51A1	Q16850	NQ	-
DDRGK1	Q96HY6	Q	NA
DHX32	Q7L7V1	NQ	-
DICER1	Q9UPY3	NQ	-
DNAJC7	Q99615	Q	NA
EPHX1	P07099	NQ	-
ESYT1	Q9BSJ8	Q	NA
GUCY1B3	Q02153	NQ	-
HSP90AA1	P07900	Q	NA
HSPA4	P34932	Q	NA
HSPA8	P11142	Q	NA
LRRC1	Q9BTT6	NQ	-
METTL9	Q9H1A3	NQ	-
MTHFR	P42898	NQ	-
NOTCH1	P46531	NQ	-
OSMR	Q99650	NQ	-
PASK	Q96RG2	NQ	-
PDP2	Q9P2J9	NQ	-
PPP1R15B	Q5SWA1	NQ	-
PPP6R1	Q9UPN7	NQ	-
PPP6R3	Q5H9R7	NQ	-
PRKAR1B	P31321	NQ	-
RETSAT	Q6NUM9	NQ	-
RNASEH2A	O75792	Q	NA
SNX14	Q9Y5W7	NQ	-
SPTLC2	O15270	NQ	-
STIM2	Q9P246	NQ	-
STIP1	P31948	Q	NA
STUB1	Q9UNE7	Q	NA
STX18	Q9P2W9	NQ	-
TBK1	Q9UHD2	NQ	-
TNFRSF10A	O00220	NQ	-
TTC4	O95801	Q	NA
TUBA1A	Q71U36	NQ	-
TUBB	P07437	Q	NA
TUBB4B	P68371	Q	NA
TUBB8	**Q3ZCM7**	**Q**	**A**
UBE2I	P63279	Q	NA
UNC93B1	Q9H1C4	NQ	-

ABCA2: ATP-binding cassette sub-family A member 2. **ABCC2:** ATP-binding cassette sub-family C member 2. **ACVR2A:** activin A type IIA receptor. **ADCY3:** adenylate cyclase 3. **ATP13A3:** ATPase type 13A3. **CIB1:** calcium and integrin binding protein 1. **CLCN3:** chloride channel 3. **CYP51A1:** cytochrome P450. **DDRGK1:** DDRGK domain containing protein 1. **DHX32:** DEAH box polypeptide 32. **DICER1:** dicer 1 (ribonuclease type 3). **DNAJC7:** DnaJ (Hsp40) homolog member 7. **EPHX1:** epoxide hydrolase 1. **ESYT1:** extended synaptotagmin-like protein 1. **GUCY1B3:** Guanylate cyclase 1-β3. **HSP90AA1:** Heat shock 90 kDa protein α-A1. **HSPA4:** heat shock 70 kDa protein 4. **HSPA8:** heat shock 70 kDa protein 8. **LRRC1:** leucine rich repeat containing protein 1. **METTL9:** methyltransferase like protein 9. **MTHFR:** methylenetetrahydrofolate reductase. **NOTCH1:** notch receptor 1. **OSMR:** oncostatin M receptor. **PASK:** PAS domain containing serin/threonine kinase. **PDP2:** pyruvate dehyrogenase phosphatase catalytic subunit 2. **PPP1R15B:** protein phosphatase 1 regulatory subunit 15B. **PPP6R1:** protein phosphatase 6 regulatory subunit 1. **PPP6R3:** protein phosphatase 6 regulatory subunit 3. **PRKAR1B:** protein kinase cAMP-dependent type I-β. **RETSAT:** retinol saturase. **RNASEH2A:** ribonuclease H2 subunit A. **SNX14:** sorting nexin 14. **SPTLC2:** serine

palmitoyltransferase long chain base subunit 2. **STIM2:** stromal interaction molecule 2. **STIP1:** stress-induced phosphoprotein 1. **STUB1:** E3 ubiquitin protein ligase. **STX18:** syntaxin 18. **TBK1:** TANK-binding kinase 1. **TNFRSF10A:** tumor necrosis factor receptor superfamily member 10a. **TUBA1A:** tubulin α-1A. **TUBB:** tubulin β-1. **TUBB4B:** tubulin β-4B. **TTC4:** tetratricopeptide repeat domain 4. **UBE2I:** ubiquitin-conjugating enzyme E2I. **UNC93B1:** unc-93 homolog B1.

Supplementary Table S2. GO-terms related with proliferative and apoptotic processes altered in NOX5-β infected hCMEC/D3 cells according to Reactome® software. GO-terms are classified by the specific phenotypic alteration and cellular pathway in which they are involved.

Phenotypic alteration	Cellular pathway	GO-term nº	GO-term description
Genomic instability	Chromatin assembly	R-HSA-73886	Chromosome Maintenance
		R-HSA-4551638	SUMOylation of chromatin organization proteins
		R-HSA-157579	Telomere Maintenance
		R-HSA-180786	Extension of Telomeres
		R-HSA-171319	Telomere Extension By Telomerase
		R-HSA-2559586	DNA Damage/Telomere Stress Induced Senescence
		R-HSA-2559584	Formation of Senescence-Associated Heterochromatin Foci (SAHF)
		R-HSA-4615885	SUMOylation of DNA replication proteins
	Aberrant transcription	R-HSA-927802	Nonsense-Mediated Decay (NMD)
		R-HSA-975957	Nonsense Mediated Decay (NMD) enhanced by the Exon Junction Complex (EJC)
		R-HSA-975956	Nonsense Mediated Decay (NMD) independent of the Exon Junction Complex (EJC)
	Cell senescence and apoptosis	R-HSA-2559583	Cellular Senescence
		R-HSA-109581	Apoptosis
		R-HSA-75153	Apoptotic execution phase
		R-HSA-140342	Apoptosis induced DNA fragmentation
		R-HSA-5357801	Programmed Cell Death
General gene expression alteration	General	R-HSA-15869	Metabolism of nucleotides
		R-HSA-8953854	Metabolism of RNA
		R-HSA-211000	Gene Silencing by RNA
		R-HSA-72312	rRNA processing
		R-HSA-72306	tRNA processing
		R-HSA-72766	Translation
		R-HSA-9609507	Protein localization
	mRNA maturation and transport	R-HSA-191859	snRNP Assembly
		R-HSA-72202	Transport of Mature Transcript to Cytoplasm
		R-HSA-159231	Transport of Mature mRNA Derived from an Intronless Transcript
		R-HSA-159234	Transport of Mature mRNAs Derived from Intronless Transcripts
		R-HSA-159230	Transport of the SLBP Dependant Mature mRNA
		R-HSA-159227	Transport of the SLBP independent Mature mRNA
		R-HSA-159236	Transport of Mature mRNA derived from an Intron-Containing Transcript
	Ribosome biogenesis	R-HSA-72312	rRNA processing
		R-HSA-8868773	rRNA processing in the nucleus and cytosol
		R-HSA-6791226	Major pathway of rRNA processing in the nucleus and cytosol
		R-HSA-191859	snRNP Assembly
		R-HSA-72689	Formation of a pool of free 40S subunits
		R-HSA-72706	GTP hydrolysis and joining of the 60S ribosomal subunit
	tRNA processing	R-HSA-194441	Metabolism of non-coding RNA
		R-HSA-72306	tRNA processing
		R-HSA-6784531	tRNA processing in the nucleus
	Protein translation	R-HSA-72766	Translation
		R-HSA-72613	Eukaryotic Translation Initiation
		R-HSA-72737	Cap-dependent Translation Initiation
		R-HSA-156902	Peptide chain elongation
		R-HSA-156842	Eukariotic translation elongation
		R-HSA-72764	Eukariotic translation termination
	Protein modifications	R-HSA-9609507	Protein localization
		R-HSA-2990846	SUMOylation
		R-HSA-4570464	SUMOylation of RNA binding proteins
		R-HSA-3232142	SUMOylation of ubiquitinylation proteins
		R-HSA-3108232	SUMO E3 ligases SUMOylate target proteins
Cell proliferation	Mitosis	R-HSA-1640170	Cell cycle
		R-HSA-69278	Cell Cycle, Mitotic
		R-HSA-1852241	Organelle biogenesis and maintenance
		R-HSA-68875	Mitotic Prophase
		R-HSA-2980766	Nuclear Envelope Breakdown
		R-HSA-3301854	Nuclear Pore Complex (NPC) Disassembly
		R-HSA-380320	Recruitment of NuMA to mitotic centrosomes
		R-HSA-453274	Mitotic G2-G2/M phases
		R-HSA-69275	G2/M Transition
		R-HSA-68886	M Phase
		R-HSA-2995410	Nuclear Envelope (NE) Reassembly

Supplementary Table S3. GO-terms related with metabolic alterations in NOX5-β infected hCMEC/D3 cells according to Reactome® software. GO-terms are classified by the specific phenotypic alteration and cellular pathway in which they are involved.

Phenotypic alteration	Cellular pathway	GO-term nº	GO-term description
Cell energetics dysregulation	General	R-HSA-9711097	Cellular response to starvation
	Glucose metabolism	R-HSA-70326	Glucose metabolism
		R-HSA-71387	Metabolism of carbohydrates
		R-HSA-70171	Glycolysis
		R-HSA-70263	Gluconeogenesis
		R-HSA-1445148	Translocation of SLC2A4 (GLUT4) to the plasma membrane
		R-HSA-170822	Regulation of Glucokinase by Glucokinase Regulatory Protein
	Protein metabolism	R-HSA-9633012	Response of EIF2AK4 (GCN2) to amino acid deficiency
		R-HSA-71291	Metabolism of amino acids and derivatives
		R-HSA-9646399	Aggrephagy
	Autophagy	R-HSA-9612973	Autophagy
		R-HSA-9663891	Selective autophagy
		R-HSA-1632852	Macroautophagy
Mitochondrial metabolism	Protein synthesis	R-HSA-1852241	Organelle biogenesis and maintenance
		R-HSA-5368287	Mitochondrial translation
		R-HSA-5368286	Mitochondrial translation initiation
		R-HSA-5389840	Mitochondrial translation elongation
		R-HSA-5419276	Mitochondrial translation termination
		R-HSA-1268020	Mitochondrial protein import
	ATP synthesis	R-HSA-611105	Respiratory electron transport
		R-HSA-6799198	Complex I biogenesis
		R-HSA-1428517	The citric acid (TCA) cycle and respiratory electron transport
		R-HSA-163200	Respiratory electron transport, ATP synthesis by chemiosmotic coupling, and heat production by uncoupling proteins
		R-HSA-156827	L13a-mediated translational silencing of Ceruloplasmin expression

Supplementary Table S4. GO-terms related with cell migration in NOX5-β infected hCMEC/D3 cells according to Reactome® software. GO-terms are classified by the specific phenotypic alteration and cellular pathway in which they are involved.

Phenotypic alteration	Cellular pathway	GO-term nº	GO-term description
Cell migration	Conformational changes	R-HSA-5617833	Cilium Assembly
		R-HSA-9711097	Axon guidance
		R-HSA-373755	Semaphorin interactions
		R-HSA-399956	CRMPs in Sema3A signaling
		R-HSA-437239	Recycling pathway of L1
		R-HSA-194315	Signaling by Rho GTPases
		R-HSA-9716542	Signaling by Rho GTPases, Miro GTPases and RHOBTB3

Supplementary Table S5. E2f transcription factor isoforms expression in NOX5-β infected HAEC transcriptomic array after 12, 18 and 24 hours of infection.

			12h		18h		24h	
Gene	GeneID	Database	FC	adj.P.Val	FC	adj.P.Val	FC	adj.P.Val
E2f3	*NM_001949*	RefSeq	**1.20**	**0.0138***	**1.33**	**0.0067***	**1.34**	**0.0049***
E2f6	*NM_001278276*	RefSeq	**1.42**	**0.0002***	**1.30**	**0.0336***	**1.44**	**0.0038***
E2f7	*NM_001256371*	RefSeq	**1.64**	**p<0.0001***	**4.58**	**p<0.0001***	**5.59**	**p<0.0001***
E2f8	*NM_001256371*	RefSeq	**0.80**	**0,0011***	**0.69**	**0.0002***	**0.74**	**0.0010***

E2f3: *E2f transcription factor 3.* ***E2f6:*** *E2f transcription factor 6.* ***E2f7:*** *E2f transcription factor 7.* ***E2f8:*** *E2f transcription factor 8.*

Supplementary Table S6. Rho GTPase family members expression in NOX5-β infected HAEC transcriptomic array after 12, 18 and 24 hours of infection.

			12h		18h		24h	
**Gene**	**GeneID**	**Database**	**FC**	**adj.P.Val**	**FC**	**adj.P.Val**	**FC**	**adj.P.Val**
RND1	ENST00000548445	ENSEMBL	**8.51**	**p<0.0001***	**10.74**	**p<0.0001***	**9.53**	**p<0.0001***
RND2	NM_005440	RefSeq	1.00	0.9997	0.94	0.7630	1.00	0.9874
RND3	NM_001254738	RefSeq	**1.47**	**p<0.0001***	**1.79**	**p<0.0001***	**1.82**	**p<0.0001***

RND1: *Rho family GTPase 1.* ***RND2:*** *Rho family GTPase 2.* ***RND3:*** *Rho family GTPase 3.*
